# Assessing the Relevance of Specific Response Features in the Neural Code

**DOI:** 10.3390/e20110879

**Published:** 2018-11-15

**Authors:** Hugo Gabriel Eyherabide, Inés Samengo

**Affiliations:** 1 Department of Computer Science and Helsinki Institute for Information Technology, University of Helsinki Gustaf Hällströmin katu 2b, FI00560 Helsinki, Finland; 2 Department of Medical Physics, Centro Atómico Bariloche and Instituto Balseiro, 8400 San Carlos de Bariloche, Argentina

**Keywords:** neural code, representation, decoding, spike-time precision, discrimination, noise correlations, information theory, mismatched decoding

## Abstract

The study of the neural code aims at deciphering how the nervous system maps external stimuli into neural activity—the encoding phase—and subsequently transforms such activity into adequate responses to the original stimuli—the decoding phase. Several information-theoretical methods have been proposed to assess the relevance of individual response features, as for example, the spike count of a given neuron, or the amount of correlation in the activity of two cells. These methods work under the premise that the relevance of a feature is reflected in the information loss that is induced by eliminating the feature from the response. The alternative methods differ in the procedure by which the tested feature is removed, and the algorithm with which the lost information is calculated. Here we compare these methods, and show that more often than not, each method assigns a different relevance to the tested feature. We demonstrate that the differences are both quantitative and qualitative, and connect them with the method employed to remove the tested feature, as well as the procedure to calculate the lost information. By studying a collection of carefully designed examples, and working on analytic derivations, we identify the conditions under which the relevance of features diagnosed by different methods can be ranked, or sometimes even equated. The condition for equality involves both the amount and the type of information contributed by the tested feature. We conclude that the quest for relevant response features is more delicate than previously thought, and may yield to multiple answers depending on methodological subtleties.

## 1. Introduction

Understanding the neural code involves, among other things, identifying the relevant response features that participate in the representation of information. Different studies have proposed several candidates, for example, the spiking rate [1,2], the response latency [3], the temporal organisation of spikes [4], the amount of synchrony in a given brain area [5], the amount of correlation between the activity of different neurons [6], or the phase of the local field potential at the time of spiking [7], to cite a few. One way of evaluating the relevance of each candidate feature is to assess how much information is lost by ignoring that feature. This strategy involves the comparison of the mutual information between the stimulus and the so-called *full response* (a collection of response features including the tested one) and the same information calculated with a *reduced response*, obtained by dropping the tested feature from the full response. If the tested feature is relevant, the information encoded by the reduced response should be smaller than that of the full response.

The procedure is fairly straightforward when the response features are defined in terms of variables that take definite values in each stimulus presentation, as for example, the spike count *C* fired in a fixed time window, or the latency *L* between the stimulus and the first spike. The full response in this case is a two-component vector [C,L], the value of which is uniquely defined for each stimulus presentation—let us assume that in this example, *C* is never equal to 0, so *L* is always well defined. The reduced response is a one-component vector, either *C* or *L*, depending whether we are evaluating the relevance of the latency or the spike count, respectively. If the latency or the spike count are relevant, then the information encoded by *C* or *L*, respectively, should be smaller than that of the pair [C,L]. Throughout this paper, we often use *C* and *L* as examples of response features that take a precise value in each trial, to contrast with other features that are only defined in the whole collection of trials, as discussed below.

The method becomes more controversial when applied to response properties that can only be defined in multiple stimulus presentations, as for example, the amount of correlation in the activity of two or more neurons, or the temporal precision of the elicited spikes. These properties cannot be calculated from single responses, so more sophisticated methods are required to delete the tested feature. There are several alternative procedures to perform such deletion, and several are also the ways in which the lost information can be calculated. Interestingly, the lost information depends markedly on the chosen method, implying that the so-called *relevance* of a given feature is a subtle concept, that needs to be specified precisely. When assessing the relevance of noise correlations, two different sets of strategies have been proposed by the seminal works of Nirenberg et al. [8] and Schneidman et al. [9]. The first proposal evaluated the role of noise correlations in *decoding* the information represented in neural activity, whereas the second, in the amount of *encoded* information. Quite surprisingly, the contribution of correlations to the decoded information was shown to sometimes exceed the amount of encoded information [9], seemingly contradicting the intuitive idea that the encoded information constitutes an upper bound to the decoded information. The apparent inconsistency between the two measures has not been observed in later extensions of the technique, where the relevance of other response aspects was evaluated, such as spike-time precision, spike-counts or spike-onsets. Moreover, it has even been argued that the inconsistency was exclusively observed when assessing the role of noise correlations [10,11,12,13].

In this paper, for the first time, the different methods used in the literature to delete a given response feature are distinguished, and the implications of each method are discussed and compared. We show that the data processing inequality, stating that the decoded information cannot surpass the encoded information, can only be invoked with some - and not all - deletion procedures. The distinction between such procedures allows us to identify the conditions in which the decoded information can exceed the encoded information, and to demonstrate that there was no logical inconsistency in previous studies. We also show explicit examples where the decoded information surpasses the encoded information also when assessing the role of other response aspects different from noise correlations. In order to explain why such behaviours have not been identified until now, we scrutinise the arguments given in the literature to claim that only noise correlations could exhibit such syndrome. We conclude that although the measures employed to assess the relevance of individual response features initially distinguished clearly between the relevance for encoding and the relevance for decoding, this distinction was eventually lost in later modifications of the measures. By diagnosing the confusion, we prove that indeed, the response features for which the decoded information can surpass the encoded information are not restricted to noise correlations.

More generally, we discuss a wide collection of strategies employed to assess the relevance of individual response features, ranging from those encoded-oriented to those decoded-oriented. This distinction is related to the way the tested feature contributes to the performance of decoders, which can be mismatched or not. The relevance of the tested feature obtained with some of the measures is always bounded by the relevance of another measure. Yet, not all measures can be ordered hierarchically. There are examples where the relevance of a feature obtained with one method may surpass or be surpassed by the relevance of another, depending on the specific values taken by the prior stimulus probability and the conditional response probabilities. We analyse a collection of carefully chosen examples to identify the cases where this is so. In certain restricted conditions, however, the hierarchy, or even the equality, can be ensured. Here we establish these conditions by means of analytic reasoning, and discuss their implications in terms of the amount and type of information encoded by the tested feature.

We also present examples in which the measures to assess the relevance of a given feature can be used to extract qualitative knowledge about the type of information encoded by the feature. In other words, we assess not only *how much* information is encoded by an individual feature, but also *what kind* of information is provided, with respect to individual stimulus attributes. Again, we prove that the type of encoded information depends on the method employed to assess it.

Finally, given that one important property of measures of relevance hinges on whether they represent the operation of matched or mismatched decoders, we also explore the consequences of operating mismatched decoders on noisy responses, instead of real responses. We conclude that it may be possible to improve the performance of a mismatched decoder by adding noise. From the theoretical point of view, this observation underscores the fact that the conditions for optimality for matched decoders need not hold for mismatched decoders. From the practical perspective, our results open new opportunities for potentially simpler, more efficient and more resilient decoding algorithms.

In Section 2.1, we establish the notation, and we introduce some of the key concepts that will be used throughout the paper. These concepts are employed in Section 2.2 to determine the cases where the data-processing inequality can be ensured. In Section 2.3 we introduce 9 measures of feature relevance that were previously defined in the literature, and briefly discuss their meaning, similarities and discrepancies. A numeric exploration of a set of carefully chosen examples is employed in Section 2.4 to detect the pairs of measures for which no general hierarchical order exists. In Section 2.5 we discuss the consequences of employing measures that are conceptually linked to matched or mismatched decoders. Later, in Section 2.6, we explore the way in which different measures of feature relevance arrogate different qualitative meaning to the type of information encoded by the tested feature. In Section 2.7 we discuss the conditions under which encoding-oriented measures provide the same amount of information as their decoding-oriented counterparts, and also the conditions under which the equality extends also to the content of that information. Then, in Section 2.8, we observe that sometimes, mismatched decoders may improve their performance when operating upon noisy responses. We discuss some relations of our work with other approaches and to the limiting sampling problem in Section 3, and we close with a summary of the main results of the paper in Section 4.

## 2. Results

### 2.1. Definitions

#### 2.1.1. Statistical Notation

When no risk of ambiguity arises, we here employ the standard abbreviated notation of statistical inference [14], denoting random variables with letters in upper case, and their values, in lower case. For example, the symbol P(x|y) always denotes the conditional probability of the random variable *X* taking the value *x* given that the random variable *Y* takes the value *y*. This notation may lead to confusion or be inappropriate, for example, when the random variable *X* takes the value *u* given that the random variable *Y* takes the value *v*. In those cases, we explicitly indicate the random variables and their values, as for example P(X=u|Y=v).

In the study of the neural code, the relevant random variables are the stimulus *S* and the response R generated by the nervous system. In this paper, we discuss the statistics of the true responses observed experimentally, and compare them with a theoretical model that describes how responses would be, if the encoding strategy were different. To differentiate these two situations, we employ the variable $R_{\mathrm{ex}}$ for the experimental responses (the real ones), and $R_{\mathrm{su}}$ for the surrogate responses (the fictitious ones). The associated conditional probability distributions are $P_{\mathrm{ex}}\left(R_{\mathrm{ex}}=r|S=s\right)$ and $P_{\mathrm{su}}\left(R_{\mathrm{su}}=r|S=s\right)$, which are often abbreviated as $P_{\mathrm{ex}}\left(r|s\right)$ and $P_{\mathrm{su}}\left(r|s\right)$, respectively. Once these distributions are known, and given the prior stimulus probabilities P(s), the joint probabilities $P_{\mathrm{ex}}\left(r,s\right)$ and $P_{\mathrm{su}}\left(r,s\right)$ can be deduced, as well as the marginals $P_{\mathrm{ex}}\left(r\right)$ and $P_{\mathrm{su}}\left(r\right)$. When interpreting the abbreviated notation, readers should keep in mind that $P_{\mathrm{ex}}$ governs the variable $R_{\mathrm{ex}}$, and $P_{\mathrm{su}}$, $R_{\mathrm{su}}$. If a statement is made about a distribution *P* or a response variable R that has no sub-index, the argument is intended for both the real and surrogate distributions or variables.

#### 2.1.2. Encoding

The process of converting stimuli *S* into neural responses R (e.g., spike-trains, local-field potentials, electroencephalographic or other brain signals, etc.) is called “encoding” [9,15]. The encoding process is typically noisy, in the sense that repeated presentations of the same stimulus may yield different neural responses, and is characterised by the joint probability distribution P(s,r). The associated marginal probabilities are

$$\begin{array}{ccc} P(s) & = & \sum_{r}P\left(s,r\right),\\ P(r) & = & \sum_{s}P\left(s,r\right), \end{array}$$

from which the conditional response probability P(r|s)=P(s,r)/P(s), and the posterior stimulus probability P(s|r)=P(s,r)/P(r) can be defined.

The mutual information that R contains about *S* is

(1) $$I\left(S;R\right)=\sum_{s,r}P\left(s,r\right)\log_{2}\frac{P(s|r)}{P(s)}.$$

More generally, the mutual information I(S;X) about *S* contained in any random variable *X*, including but not limited to R, can be computed using the above formula with R replaced by *X*. For compactness, we denote I(S;X) as $I_{X}$ unless ambiguity arises.

#### 2.1.3. Data Processing Inequalities

When the response $R_{2}$ is a post-processed version of the response $R_{1}$, the joint probability distribution $P(s,r_{1},r_{2})$ can be written as $P\left(s,r_{1}\right)\;P\left(r_{2}|r_{1}\right)$. This decomposition implies that $R_{2}$ is conditionally independent of *S*. In these circumstances, the information about *S* contained in $R_{2}$ cannot exceed the information about *S* contained in $R_{1}$ [16]. In addition, the accuracy of the optimal decoder operating on $R_{2}$ cannot exceed the accuracy of the optimal decoder operating on $R_{1}$ [17]. These results constitute the data processing inequalities.

#### 2.1.4. Decoding

The process of transforming responses r into estimated stimuli $\hat{s}$ is called “decoding” [9,15]. More precisely, a decoder is a mapping $r\to \hat{s}$ defined by a function $\hat{s}=D\left(r\right)$. The inverse of this function is $D^{-1}$, and when *D* is not injective, $D^{-1}$ is a multi-valued mapping. The joint probability $P(s,\hat{s})$ of the presented and estimated stimuli, also called “confusion matrix” [12], is

(2) $$P\left(s,\hat{s}\right)=\sum_{r\in D^{-1}\left(\hat{s}\right)}P(s,r),$$

where the sum runs over all responses r that are mapped onto $\hat{s}$ by *D*. The information that $\hat{S}$ preserves about *S* is $I_{\hat{S}}$, and can be calculated from the confusion matrix of Equation (2). The decoding accuracy above chance level is here defined as

(3) $$A=\sum_{s}P(S=s,\hat{S}=s)-\max_{s}P(s).$$

#### 2.1.5. Optimal Decoding

Although all mappings *D* are formally admissible as decoders, not all are useful. The aim of a decoder is to make a good guess of the external stimulus *S* from the neural response R. It is therefore important to be able to construct decoders that make good guesses, or at least, as good as the mapping from stimuli to responses allows. Optimal decoders (also called *Bayesian* or *maximum-a-posteriori* decoders, as well as *ideal homunculus*, or *observer*, among other names) are defined as [18,19]

(4) $$\hat{s}=D_{\mathrm{opt}}\left(r\right)=\arg\max_{s}P(s|r)=\arg\max_{s}P(s,r).$$

This mapping selects, for each response r, the stimulus $\hat{s}$ that most likely generated r. It is optimal in the sense that any other decoding algorithm yields a confusion matrix with lower decoding accuracy. Equation (4) depends on P(s,r), so the decoder cannot be defined before knowing the functional shape of the joint probability distribution between stimuli and responses. The process of estimating P(s,r) from real data, and the subsequent insertion of the obtained distribution in Equation (4) is called the *training* of the decoder. The word “training” makes reference to a gradual process, originally stemming from a computational strategy employed to estimate the distribution progressively, while the data was being gathered. However, in this paper we do not discuss estimation strategies from limited samples, so for us, “training a decoder” is equivalent to constructing a decoder from Equation (4).

#### 2.1.6. Extensions of Optimal Decoding

The study of Ince et al. [20] introduced the concept of ranked decoding, in which each response r is mapped onto a list of *K* stimuli $\hat{s}=\left({\hat{s}}_{1},\ldots ,{\hat{s}}_{K}\right)$ ordered according to their posterior probabilities so that $P\left({\hat{s}}_{k}|r\right)\geq P\left({\hat{s}}_{k+1}|r\right)$ (with 1≤k<K, and K≤ the total number of stimuli in the experiment). Ranked decoding can provide useful models for intermediate stages in the decision pathway, and the information loss induced by ranked decoding was computed recently [17]. The joint probability associated with ranked decoding is

(5) $$P\left(s,\hat{s}\right)=\sum_{r\in D^{-1}\left(\hat{s}\right)}P(s,r),$$

where the sum runs over all response vectors r that produce the same ranking $\hat{s}$. Although $P(s,\hat{s})$ can be used to compute the information $I_{\hat{S}}$ between *S* and $\hat{S}$, it cannot be used to compute the decoding accuracy above chance level because the support of $\hat{S}$ (i.e., the set of stimulus lists) is not contained in the support of *S* (i.e., the set of stimuli).

#### 2.1.7. Approximations to Optimal Decoding

For given probabilities P(r|s) and P(s), Equation (4) defines a mapping between each response r and a candidate stimulus $\hat{s}$. In the study of the neural code, scientists often wonder what would happen if responses were not governed by the experimentally recorded distribution $P_{\mathrm{ex}}\left(r|s\right)$, but by some other surrogate distribution $P_{\mathrm{su}}\left(r|s\right)$. If we replace $P_{\mathrm{ex}}\left(r|s\right)$ by $P_{\mathrm{su}}\left(r|s\right)$ in Equation (4), we define a new decoding algorithm

(6) $$\hat{s}=D_{\mathrm{su}}\left(r\right)=\arg\max_{s}P_{\mathrm{su}}\left(s|r\right)=\arg\max_{s}P_{\mathrm{su}}\left(s,r\right).$$

which, as discussed below, may or may not be optimal, depending on how the decoder is used.

#### 2.1.8. Two Different Decoding Strategies

One alternative, here referred to as “decoding method α” is that, for each response r obtained experimentally, one decodifies a stimulus $\hat{s}$ using the new mapping of Equation (6). In this case, the chain $s\to r\to \hat{s}$ gives rise to the confusion matrix

(7) $$P^{\alpha }\left(s,\hat{s}\right)=\sum_{r\in D_{\mathrm{su}}^{-1}\left(\hat{s}\right)}P_{\mathrm{ex}}\left(s,r\right),$$

where the sum runs over all response vectors r that are mapped onto $\hat{s}$ by the new decoding algorithm $D_{\mathrm{su}}$, and the probability $P_{\mathrm{ex}}\left(r,s\right)$ appearing in the right-hand side is the real one, since responses r are generated experimentally. It is easy to see that in this case, the decoding accuracy of the new algorithm is suboptimal, since responses r are generated with the original distribution $P_{\mathrm{ex}}\left(r|s\right)$, and for that distribution, the optimal decoder is given by Equation (4) with $P=P_{\mathrm{ex}}$. In the literature, training a decoder with a probability $P_{\mathrm{su}}\left(r|s\right)$ and then operating it on variables that are generated with $P_{\mathrm{ex}}\left(r|s\right)$ is called *mismatched decoding*. In what follows, information values calculated from the distribution of Equation (7) are noted as $I_{\hat{S}}^{\alpha }$.

A second alternative, “decoding method β,” is that, for each stimulus *s*, a surrogate response $R_{\mathrm{su}}$ is drawn using the new distribution $P_{\mathrm{su}}\left(r|s\right)$. If the sampled value is $R_{\mathrm{su}}=r$, the stimulus $\hat{s}=D_{\mathrm{su}}\left(r\right)$ is decoded. In this case, the confusion matrix is

(8) $$P^{\beta }\left(s,\hat{s}\right)=\sum_{r\in D_{\mathrm{su}}^{-1}\left(\hat{s}\right)}P_{\mathrm{su}}\left(s,r\right),$$

where as before, the sum runs over all response vectors r that are mapped onto $\hat{s}$ by the decoding algorithm $D_{\mathrm{su}}\left(r\right)$, but now the probability $P_{\mathrm{su}}\left(r,s\right)$ appearing in the right-hand side is the surrogate one, since responses $R_{\mathrm{su}}$ are not generated experimentally. In this case, there is no mismatch between the construction and operation of the decoder, and $D_{\mathrm{su}}$ is optimal, in the sense that no other algorithm decodes $R_{\mathrm{su}}$ with higher decoding accuracy. One should bear in mind, however, that the surrogate responses are not the responses observed experimentally, that they may well take values in a response set that does not coincide with the set of real responses, and that $R_{\mathrm{su}}$ is not necessarily obtained by transforming the real response $R_{\mathrm{ex}}$ with a stimulus-independent mapping (see below). In what follows, information values calculated from the distribution of Equation (8) are noted as $I_{\hat{S}}^{\beta }$. Methods α and β can be easily extended to encompass also ranked decoding, *mutatis mutandis*.

The two alternative decoding methods yield two different decoding accuracies. To distinguish them, we use the notation $A_{R_{1}}^{R_{2}}$. The superscript indicates the variable whose probability distribution is used to construct the decoder in Equation (4), and consequently, determines the set of $r\in D_{\mathrm{su}}^{-1}\left(\hat{s}\right)$ that contribute to the sums of Equations (7) and (8). The subscript indicates the variable upon which the decoder is applied, and its probability distribution is summed in the right-hand side of Equations (7) and (8). That is, $A_{R_{1}}^{R_{2}}$ is computed through Equation (3) with

(9) $$P_{R_{1}}^{R_{2}}\left(s,\hat{s}\right)=\sum_{r\in D_{R_{2}}^{-1}\left(\hat{s}\right)}P(S=s,R_{1}=r),$$

so that $P^{\alpha }\left(s,\hat{s}\right)=P_{R_{\mathrm{ex}}}^{R_{\mathrm{su}}}\left(s,\hat{s}\right)$ and $P^{\beta }\left(s,\hat{s}\right)=P_{R_{\mathrm{su}}}^{R_{\mathrm{su}}}\left(s,\hat{s}\right)$.

### 2.2. The Applicability of the Data-Processing Inequality

Assessing the relevance of a response feature typically involves a subtraction $\Delta I=I-I^{\prime }$, where *I* and $I^{\prime }$ represent the mutual information between stimuli and a set of response features containing or not containing the tested feature, respectively. The magnitude of ΔI is often interpreted as the information provided by the tested feature. This interpretation requires ΔI to be positive, since intuitively, one would imagine that removing a response feature cannot increase the encoded information. As shown below, a formal proof of this intuition may or may not be possible invoking the data processing inequality (see Section 2.1.3 and reference [16]), depending on the method used to eliminate the tested feature. As a consequence, there are cases in which ΔI is indeed negative (see below). In these cases, the tested feature is detrimental to information encoding [9].

#### 2.2.1. Reduced Representations

There are several procedures by which the tested feature can be removed from the response. The validity of the data-processing inequalities (see definition in Section 2.1.3) depends on the chosen procedure. In order to specify the conditions in which the inequalities hold, we here introduce the concept of *reduced representations*. When the response feature under evaluation is removed from $R_{\mathrm{ex}}$ by a deterministic mapping $R_{\mathrm{su}}=f\left(R_{\mathrm{ex}}\right)$, we call the obtained variable $R_{\mathrm{su}}$ a *reduced representation* of $R_{\mathrm{ex}}$. A required condition for a mapping to be a reduced representation is that the function *f* be stimulus-independent, that is, that the value of $R_{\mathrm{su}}$ be conditionally independent from *s*. Mathematically, this means that $P\left(r_{\mathrm{su}},s|r_{\mathrm{ex}}\right)=P\left(r_{\mathrm{su}}|r_{\mathrm{ex}}\right)P\left(s|r_{\mathrm{ex}}\right)$. If the mapping *f* and the conditional response distribution $P_{\mathrm{ex}}\left(r|s\right)$ are known, the distribution $P_{\mathrm{su}}\left(r|s\right)$ can be derived using standard methods. The data processing inequality ensures that for all reduced representations, $I_{R_{\mathrm{ex}}}\geq I_{R_{\mathrm{su}}}$.

Reduced representations are usually employed when the response feature whose relevance is to be assessed takes a definite value in each trial, as happens for example, with the number of spikes in a fixed time window, the latency of the firing response, or the activity of a specific neuron in a larger population of neurons. In these cases it is easy to construct $R_{\mathrm{su}}$ simply by dropping from $R_{\mathrm{ex}}$ the tested feature, or by fixing its value with some deterministic rule.

**Figure 1:**
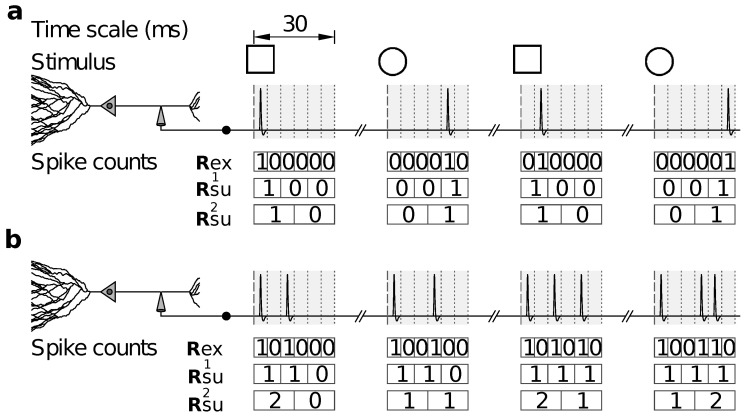
Assessing the relevance of response accuracy by varying the duration of the temporal bin. (**a**) Hypothetical intracellular recording of the spike patterns elicited by a single neuron after presenting in alternation two visual stimuli, □ and ◯, each of which triggers two possible responses displayed in columns 1 and 3 for □, and 2 and 4 for ◯. Stimulus probabilities and conditional response probabilities are arbitrary. Time is discretized in bins of 5 ms. The responses are recorded within 30 ms time-windows after stimulus onset. Spikes are fired with latencies that are uniformly distributed between 0 and 10 ms after the onset of □, and between 20 and 30 ms after the onset of ◯. Responses are represented by counting the number of spikes within consecutive time-bins of size 5, 10 and 15 ms starting from stimulus onset, thereby yielding discrete-time sequences $R_{\mathrm{ex}}$, $R_{\mathrm{su}}^{1}$ and $R_{\mathrm{su}}^{2}$, respectively; (**b**) Same as a, but with stimuli producing two different types of response patterns composed of 2 or 3 spikes.

Reduced representations can also be used in other cases, for example, when the relevance of the feature *response accuracy* is assessed. This feature does not take a specific value in each trial; only by comparing multiple trials can the response accuracy be determined. A widely-used strategy is to represent spike trains with temporal bins of increasing duration, and to evaluate how the amount of information decreases as the representation becomes coarser. A sequence of surrogate responses is thereby defined, by progressively disregarding the fine temporal precision with which spike trains were recorded (Figure 1).

Several studies have reported an information $I_{R_{\mathrm{su}}}$ that decreases monotonically with the duration δt of the time bin (for example [21,22,23]). If there is a specific temporal scale in which spike-time precision is relevant—the alleged argument goes—a sudden drop in $I_{R_{\mathrm{su}}}\left(\delta t\right)$ appears at the relevant scale. It should be noted, however, that the data processing inequality does not ensure that $I_{R_{\mathrm{su}}}\left(\delta t\right)$ be a monotonically decreasing function of δt. In the example of Figure 1, representations $R_{\mathrm{su}}^{1}$ and $R_{\mathrm{su}}^{2}$ are defined with long temporal bins, the durations of which are integer multiples of the bin used for $R_{\mathrm{ex}}$. Hence, $R_{\mathrm{su}}^{1}$ and $R_{\mathrm{su}}^{2}$ are reduced representations of $R_{\mathrm{ex}}$, and the data processing inequality does indeed guarantee that $I_{R_{\mathrm{ex}}}\geq I_{R_{\mathrm{su}}^{1}}$ and $I_{R_{\mathrm{ex}}}\geq I_{R_{\mathrm{su}}^{2}}$. However, $R_{\mathrm{su}}^{2}$ is not a reduced representation of $R_{\mathrm{su}}^{1}$, so there is no reason why $I_{R_{\mathrm{su}}^{2}}$ should be smaller than $I_{R_{\mathrm{su}}^{1}}$, and indeed, Figure 1b shows an example where it is not. The representation constructed with bins of intermediate duration, namely 10 ms, does not distinguish between the two stimuli, whereas those of shorter and longer duration, 5 and 15 ms, do. A similar effect can be observed in the experimental data (freely available online) of Lefebvre et al. [24], when analysed with bins of sizes 5, 10 and 15 ms in windows of total duration 60 ms. Although these examples are rare, they demonstrate that there is no theoretical substantiation to the expectation of $I_{R_{\mathrm{su}}}$ to drop monotonically with increasing δt.

#### 2.2.2. Stochastically Reduced Representations

When the response feature under evaluation is removed from the response variable $R_{\mathrm{ex}}$ by a stochastic mapping $R_{\mathrm{ex}}\to R_{\mathrm{su}}$, the obtained variable $R_{\mathrm{su}}$ is called a *stochastically reduced representation* of $R_{\mathrm{ex}}$. A required condition for a mapping to be a stochastically reduced representation is that the probability distribution of each $R_{\mathrm{su}}$ be dependent on $R_{\mathrm{ex}}$, but conditionally independent from *s*. In these circumstances, the data processing inequality ensures that $I_{R_{\mathrm{ex}}}\geq I_{R_{\mathrm{su}}}$. If the statistical properties of the noisy components of the mapping are known, as well as the conditional response probability distribution $P_{\mathrm{ex}}\left(r|s\right)$, the distribution $P_{\mathrm{su}}\left(r|s\right)$ can be derived using standard methods. Formally, stochastic representations $R_{\mathrm{su}}$ are obtained through stimulus-independent stochastic functions of the original representation $R_{\mathrm{ex}}$. After observing that $R_{\mathrm{ex}}$ adopted the value $r_{\mathrm{ex}}$, these functions produce a single value $r_{\mathrm{su}}$ for $R_{\mathrm{su}}$ chosen with transition probabilities $Q(r_{\mathrm{su}}|r_{\mathrm{ex}})$ such that

(10) $$P_{\mathrm{su}}\left(r_{\mathrm{su}}|s\right)=\sum_{r_{\mathrm{ex}}}P_{\mathrm{ex}}\left(r_{\mathrm{ex}}|s\right)Q\left(r_{\mathrm{su}}|r_{\mathrm{ex}}\right).$$

**Figure 2:**
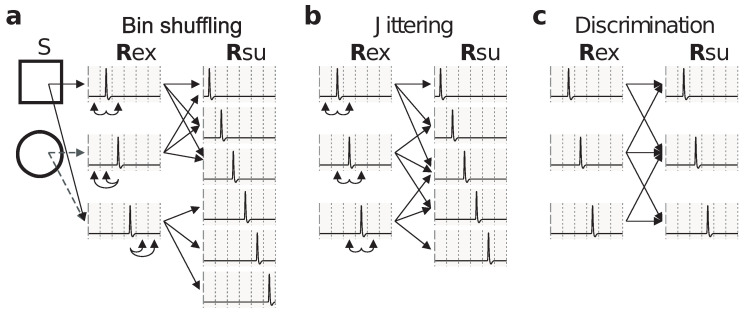
Examples of stochastic codes. Alternative ways of assessing the relevance of spike-timing precision. (**a**) Stochastic function (arrows on the left) modeling the encoding process. The elicited response $r_{\mathrm{ex}}$ is turned into a surrogate response $r_{\mathrm{su}}$ with a transition probability $Q(r_{\mathrm{su}}|r_{\mathrm{ex}})$ given by Equation (11). This function turns $R_{\mathrm{ex}}$ into a stochastic representation $R_{\mathrm{su}}$ by shuffling spikes and silences within bins of 15ms starting from stimulus onset; (**b**) Responses $r_{\mathrm{ex}}$ in panel (a) are transformed by a stochastic function with $Q(r_{\mathrm{su}}|r_{\mathrm{ex}})$ given by Equation (12), which introduces jitter uniformly distributed within 15ms windows centered at each spike; (**c**) Responses $r_{\mathrm{ex}}$ in panel (a) are transformed by a stochastic function with $Q(r_{\mathrm{su}}|r_{\mathrm{ex}})$ given by Equation (13), which models the inability to distinguish responses with spikes occurring in adjacent bins, or equivalently, with distances $d^{\mathrm{spike}}\left[q=1\right]\leq 1$ or $d^{\mathrm{interval}}\left[q=1\right]\leq 1$ (see [25,26] for further remarks on these distances). Notice that $R_{\mathrm{su}}$ samples the same response set as $R_{\mathrm{ex}}$.

To illustrate the utility of stochastically reduced representations, we discuss their role in providing alternative strategies when assessing the relevance of spike-timing precision, not by changing the bin size as in Figure 1, but by randomly manipulating the responses, as illustrated in Figure 2.

The method of Figure 2a yields the same information $I_{R_{\mathrm{su}}}$ and response accuracy as the method producing $R_{\mathrm{su}}^{2}$ in Figure 1. Each method yields responses that can be related to the responses of the other method through a stimulus-independent deterministic or stochastic function. Both methods suffer from the same drawback: They treat spikes differently depending on their location within the 15 ms time window. Indeed, both methods preserve the distinction between two spikes located in different windows, but not within the same window, even if the separation between the spikes is the same. The mapping illustrated in Figure 2a has transition probabilities

(11) $$Q\left(r_{\mathrm{su}}|r_{\mathrm{ex}}\right)=\frac{1}{3}\left[\begin{array}{cccccc} 1 & 1 & 1 & 0 & 0 & 0\\ 1 & 1 & 1 & 0 & 0 & 0\\ 0 & 0 & 0 & 1 & 1 & 1 \end{array}\right],$$

where rows enumerate the elements of the ordered set $R_{ex}=\left\{\left[2\right],\left[3\right],\left[4\right]\right\}$ from where $R_{\mathrm{ex}}$ is sampled, and columns enumerate the elements of the ordered set $R_{su}=\left\{\left[1\right],\left[2\right],\left[3\right],\left[4\right],\left[5\right],\left[6\right]\right\}$ from where $R_{\mathrm{su}}$ is sampled.

A third method, jittering, consists in shuffling the recorded spikes within time windows centered at each spike (Figure 2b). The responses generated by this method need not be obtainable from the responses generated by the mappings of Figure 2a or Figure 1 through stimulus-independent stochastic functions. Still, the method of Figure 2b inherently yields a stochastic code, and, unlike the methods discussed previously, treats all spikes in the same manner. The mapping illustrated in Figure 2b has transition probabilities

(12) $$Q\left(r_{\mathrm{su}}|r_{\mathrm{ex}}\right)=\frac{1}{3}\left[\begin{array}{ccccc} 1 & 1 & 1 & 0 & 0\\ 0 & 1 & 1 & 1 & 0\\ 0 & 0 & 1 & 1 & 1 \end{array}\right],$$

where rows enumerate the elements of the ordered set $R_{ex}=\left\{\left[2\right],\left[3\right],\left[4\right]\right\}$ from where $R_{\mathrm{ex}}$ is sampled, and columns enumerate the elements of the ordered set $R_{su}=\left\{\left[1\right],\left[2\right],\left[3\right],\left[4\right],\left[5\right]\right\}$ from where $R_{\mathrm{su}}$ is sampled.

As a fourth example, consider the effect of response discrimination, as studied in the seminal work of Victor and Purpura [25]. There, two responses were considered indistinguishable when some measure of distance between the responses was less than a predefined threshold. However, neural responses were transformed through a method based on cross-validation that is not guaranteed to be stimulus-independent. Depending on the case, hence, this fourth method may or may not be a stochastically reduced representation. The case chosen in Figure 2c is a successful example, and the associated matrix of transition probabilities is

(13) $$Q\left(r_{\mathrm{su}}|r_{\mathrm{ex}}\right)=\frac{1}{6}\left[\begin{array}{ccc} 3 & 3 & 0\\ 2 & 2 & 2\\ 0 & 3 & 3 \end{array}\right],$$

where rows and columns enumerate the elements of the ordered set $R_{ex}=R_{su}=\left\{\left[2\right],\left[3\right],\left[4\right]\right\}$ from where both $R_{\mathrm{ex}}$ and $R_{\mathrm{su}}$ are sampled.

Other methods exist which merge indistinguishable responses, thereby yielding reduced representations. These methods, however, are limited to notions of similarity that are transitive, a condition not fulfilled, for example, by those based on Euclidean distance, edit distance, or by the case of Figure 2c.

**Figure 3:**
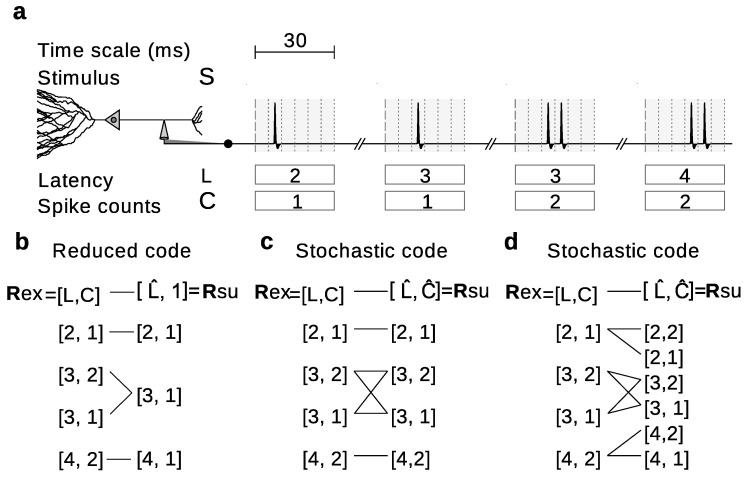
Stochastically reduced representations include and generalize deterministically reduced representations. (**a**) Analogous description to Figure 1a, but with responses characterized using a representation $R_{\mathrm{ex}}=\left[L,C\right]$ based on the first-spike latency (*L*) and the spike-count (*C*); (**b**) Deterministic transformation (arrows) of $R_{\mathrm{ex}}$ in panel a into a reduced code $R_{\mathrm{su}}=\left[\hat{L},1\right]$, which ignores the additional information carried in *C* by considering it constant and equal to unity. This reduced code can also be reinterpreted as a stochastic code with transition probabilities $Q(r_{\mathrm{su}}|r_{\mathrm{ex}})$ defined by Equation (14); (**c**) The additional information carried in *C* is here ignored by shuffling the values of *C* across all trails with the same *L*, thereby turning $R_{\mathrm{ex}}$ in panel a into a stochastic code $R_{\mathrm{su}}=\left[\hat{L},\hat{C}\right]$ with transition probabilities $Q(r_{\mathrm{su}}|r_{\mathrm{ex}})$ defined by Equation (15); (**d**) The additional information carried in *C* is here ignored by replacing the actual value of *C* for one chosen with some possibly *L*-dependent probability distribution (Equation (16)).

Stochastically reduced representations include reduced representations as limiting cases. Indeed, when for each $r_{\mathrm{ex}}$ there is a $r_{\mathrm{su}}$ such that $Q(r_{\mathrm{su}}|r_{\mathrm{ex}})=1$, stochastic representations become reduced representations (Figure 3). The possibility to include stochasticity, however, broadens the range of alternatives. Consider for example the hypothetical experiment in Figure 3a, in which the neural responses $R_{\mathrm{ex}}=\left[L,C\right]$ can be completely characterized by the first-spike latencies (*L*) and the spike counts (*C*). The importance of *C* can be studied for example by using a reduced code that replaces all *C*-values with a constant (Figure 3b). In this case,

(14) $$Q\left(r_{\mathrm{su}}|r_{\mathrm{ex}}\right)=\left[\begin{array}{ccc} 1 & 0 & 0\\ 0 & 1 & 0\\ 0 & 1 & 0\\ 0 & 0 & 1 \end{array}\right],$$

where rows enumerate the elements of the ordered set $R_{ex}=\left\{\left[2,1\right],\left[3,1\right],\left[3,2\right],\left[4,2\right]\right\}$ from where $R_{\mathrm{ex}}$ is sampled, and columns enumerate the elements of the ordered set $R_{su}=\left\{\left[2,1\right],\left[3,1\right],\left[4,1\right]\right\}$ from where $R_{\mathrm{su}}$ is sampled.

Another alternative is to assess the relevance of *C* by means of a stochastic code that shuffles the values of *C* across all responses with the same *L* (Figure 3c). In this case,

(15) $$Q\left(r_{\mathrm{su}}|r_{\mathrm{ex}}\right)=\left[\begin{array}{cccc} 1 & 0 & 0 & 0\\ 0 & a & \bar{a} & 0\\ 0 & a & \bar{a} & 0\\ 0 & 0 & 0 & 1 \end{array}\right]$$

where rows enumerate the elements of the ordered set $R_{ex}=\left\{\left[2,1\right],\left[3,1\right],\left[3,2\right],\left[4,2\right]\right\}$ from where $R_{\mathrm{ex}}$ is sampled, and columns enumerate the elements of the ordered set $R_{su}=\left\{\left[2,1\right],\left[3,1\right],\left[3,2\right],\left[4,2\right]\right\}$ from where $R_{\mathrm{su}}$ is sampled. The parameter *a* is arbitrary, as long as 0<a<1. We use the notation $\bar{a}=1-a$.

A third option is to use a stochastic code that preserves the original value of *L* but chooses the value of *C* from some possibly L-dependent probability distribution (Figure 3d), for which

(16) $$Q\left(r_{\mathrm{su}}|r_{\mathrm{ex}}\right)=\left[\begin{array}{cccccc} b & 0 & 0 & \bar{b} & 0 & 0\\ 0 & c & 0 & 0 & \bar{c} & 0\\ 0 & c & 0 & 0 & \bar{c} & 0\\ 0 & 0 & d & 0 & 0 & \bar{d} \end{array}\right]$$

where rows enumerate the elements of the ordered set $R_{ex}=\left\{\left[2,1\right],\left[3,1\right],\left[3,2\right],\left[4,2\right]\right\}$ from where $R_{\mathrm{ex}}$ is sampled, and columns enumerate the elements of the ordered set $R_{su}=\left\{\left[2,1\right],\left[3,1\right],\left[4,1\right],\left[2,2\right],\left[3,2\right],\left[4,2\right]\right\}$ from where $R_{\mathrm{su}}$ is sampled. The parameters a,b,c and *d* are arbitrary, as long as 0<a,b,c,d<1; and we have used the notation $\bar{x}=1-x$ for any number *x*.

#### 2.2.3. Modification of the Conditional Response Probability Distribution

When the response feature under evaluation is removed by altering the real conditional response probability distribution $P_{\mathrm{ex}}\left(r|s\right)$, and transforming it into a surrogate distribution $P_{\mathrm{su}}\left(r|s\right)$, the obtained response model is here said to implement a *probabilistic removal* of the tested feature. Probabilistic removals are usually employed when assessing the relevance of correlations between neurons in a population, since correlations are not a variable that can be deleted from each individual response. For example, if $R=(R_{1},\ldots ,R_{n})$ represents the spike count of *n* different neurons, the real distribution $P_{\mathrm{ex}}\left(r_{1},\ldots ,r_{n}|s\right)$ is replaced by a new distribution $P_{\mathrm{su}}\left(r_{1},\ldots ,r_{n}|s\right)$ in which all neurons are conditionally independent, that is,

(17) $$P_{\mathrm{su}}\left(r|s\right)=P_{NI}\left(r|s\right)=\prod_{i=1}^{n}P_{\mathrm{ex}}\left(r_{i}|s\right),$$

where, following the notation introduced previously [17], the generic subscript “su” was replaced by “NI” to indicate “noise-independent”.

The probabilistic removal of a response feature may or may not be describable in terms of a deterministically or a stochastically reduced representation. In other words, there may or may not exist a mapping $R_{\mathrm{ex}}\to R_{\mathrm{su}}$, or equivalently, a matrix of transition probabilities $Q(r_{\mathrm{su}}|r_{\mathrm{ex}})$, that captures the replacement of $P_{\mathrm{ex}}\left(r|s\right)$ by $P_{\mathrm{su}}\left(r|s\right)$. It is important to assess whether such a matrix exists, since the data processing inequality is only guaranteed to hold with reduced representations, stochastic or not. If no reduced representation can capture the effect of a probabilistic removal, the data processing inequality may not hold, and $I_{R_{\mathrm{su}}}$ may well be larger than $I_{R_{\mathrm{ex}}}$.

In order to determine whether a stochastically reduced representation exists, the first step is to discern whether Equation (10) constitutes a compatible or an incompatible linear system for the matrix elements $Q(r_{\mathrm{su}}|r_{\mathrm{ex}})$. If the system is incompatible, there is no solution. In the compatible case, which is often indeterminate, a solution entirely composed of non-negative numbers that sum up to unity in each row is required. Given enough time and computational power, the problem can always be solved in the framework of linear programming [27]. In practical cases, however, the search is often hampered by the curse of dimensionality. To facilitate the labour, here we list a few necessary (though not sufficient) conditions that must be fulfilled for the mapping to exist. If any of the following properties does not hold, Equation (10) has no solution, so there is no need to begin a search.

**Property** **1.** *Let μ(s) be a probability distribution defined in the set of stimuli that may or may not be equal to the actual distribution with which stimuli appear in the experiment under study. For any stimulus s, the inequality $I_{\mu }\left(R_{\mathrm{su}};S=s\right)\leq I_{\mu }\left(R_{\mathrm{ex}};S=s\right)$ between stimulus-specific informations [28,29] must hold, where*

(18) $$I_{\mu }\left(R;S=s\right)=\sum_{r}P\left(r|s\right)\log_{2}\frac{P(r|s)}{\sum_{s^{\prime }}P\left(r|s^{\prime }\right)\mu \left(s^{\prime }\right)}.$$

**Proof.**  If $Q(r_{\mathrm{su}}|r_{\mathrm{ex}})$ exists, then Equation (10) can be inserted in Equation (18). Using the log-sum inequality [16], Property 1 follows. □

If we multiply both sides of the inequality by $\mu (s^{\prime })$ and sum over $s^{\prime }$, we obtain an inequality between the mutual informations $I_{\mu }\left(R_{\mathrm{su}};S\right)\leq I_{\mu }\left(R_{\mathrm{ex}};S\right)$. If μ(s)=P(s), this result reduces to the data-processing inequality $I_{R_{\mathrm{su}}}\leq I_{R_{\mathrm{ex}}}$.

**Property** **2.** *If $Q(r_{\mathrm{su}}|r_{\mathrm{ex}})$ exists, then $Q(r_{\mathrm{su}}|r_{\mathrm{ex}})=0$ whenever $P_{\mathrm{ex}}\left(s,r_{\mathrm{ex}}\right)>0$ and $P_{\mathrm{su}}\left(s,r_{\mathrm{su}}\right)=0$ for at least some s*.

**Proof.**  Suppose that $Q(r_{\mathrm{su}}|r_{\mathrm{ex}})>0$ when $P_{\mathrm{ex}}\left(s,r_{\mathrm{ex}}\right)>0$ for some *s*. Then, Equation (10) yields $P_{\mathrm{su}}\left(r_{\mathrm{su}}|s\right)>0$, contradicting the hypothesis that $P_{\mathrm{su}}\left(r_{\mathrm{su}}|s\right)=0$. Hence, $Q(r_{\mathrm{su}}|r_{\mathrm{ex}})$ must vanish. □

**Figure 4:**
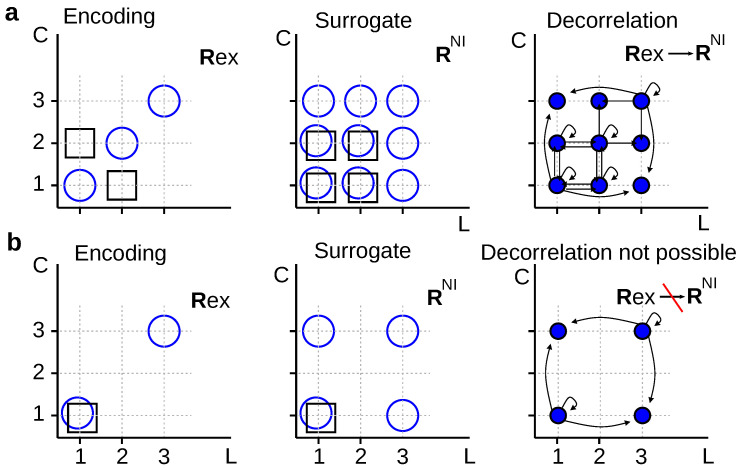
Relation between probabilistic removal and stochastic codes. (**a**) Cartesian coordinates depicting: on the left, responses $R_{\mathrm{ex}}$ of a neuron for which *L* and *C* are positively correlated when elicited by ◯, and negatively correlated when elicited by □; in the middle, the surrogate responses $R_{\mathrm{su}}=R_{NI}$ that would occur should *L* and *C* be noise independent (middle); and on the right, a stimulus-independent stochastic function that turns $R_{\mathrm{ex}}$ into $R_{\mathrm{su}}$ with $Q(r_{\mathrm{su}}|r_{\mathrm{ex}})$ given by Equation (19); (**b**) Same description as in (**a**), but with *L* and *C* noise independent given □, and with the stochastic function depicted on the right turning $R_{\mathrm{ex}}$ into $R_{NI}$ given ◯ but not □.

For example, in Figure 4a, we decorrelate first-spike latencies (*L*) and spike counts (*C*) by replacing the true conditional distribution $P_{\mathrm{ex}}\left(r|s\right)$ (left panel) by its noise-independent version $P_{\mathrm{su}}=P_{NI}\left(r|s\right)$ defined in Equation (17) (middle panel). Before searching for a mapping $R_{\mathrm{ex}}\to R_{\mathrm{su}}$, we verify that the condition $I_{R_{\mathrm{ex}}}>I_{R_{\mathrm{su}}}$ holds. Moreover, for several choices of μ(◯) and μ(□), one may confirm that $I_{\mu }\left(R_{\mathrm{ex}};S=◯\right)>I_{\mu }\left(R_{\mathrm{su}};S=◯\right)$, as well as $I_{\mu }\left(R_{\mathrm{ex}};S=□\right)>I_{\mu }\left(R_{\mathrm{su}};S=□\right)$. These results motivate the search for a solution of Equation (10) for $Q(r_{\mathrm{su}}|r_{\mathrm{ex}})$. The transition probability must be zero at least whenever $R_{\mathrm{su}}\in \left\{\left[1,3\right];\left[2,3\right];\left[3,3\right];\left[3,2\right];\left[3,1\right]\right\}$ and $R_{\mathrm{ex}}\in \left\{\left[1,2\right];\left[2,1\right]\right\}$ (Property 2). One possible solution is

(19) $$Q\left(r_{\mathrm{su}}|r_{\mathrm{ex}}\right)=\frac{1}{2}\left[\begin{array}{ccccccccc} 2b & \bar{b}c & \bar{c}\bar{b} & \bar{b}c & 0 & 0 & \bar{c}\bar{b} & 0 & 0\\ \bar{a} & 2a & 0 & 0 & \bar{a} & 0 & 0 & 0 & 0\\ a & 0 & 0 & 2\bar{a} & a & 0 & 0 & 0 & 0\\ 0 & b & 0 & b & 2\bar{b}c & \bar{c}\bar{b} & 0 & \bar{c}\bar{b} & 0\\ 0 & 0 & b & 0 & 0 & \bar{b}c & b & \bar{b}c & 2\bar{c}\bar{b} \end{array}\right].$$

where each response is defined by a vector [L,C], and rows and columns enumerate the elements of the ordered sets $R_{ex}=\left\{\left[1,1\right],\left[1,2\right],\left[2,1\right],\left[2,2\right],\left[3,3\right]\right\}$ and $R_{su}=\left\{\left[1,1\right],\left[1,2\right],\left[1,3\right],\left[2,1\right],\left[2,2\right],\left[2,3\right],\left[3,1\right],\left[3,2\right],\left[3,3\right]\right\}$ from where $R_{\mathrm{ex}}$ and $R_{\mathrm{su}}$ are sampled, respectively. In Equation (19), $a=P_{\mathrm{ex}}\left(\left[1,2\right]|□\right)$; $b=P_{\mathrm{ex}}\left(\left[1,1\right]|◯\right)$; and $c=P_{\mathrm{ex}}\left(\left[2,2\right]|◯\right)/\bar{b}$.

However, stochastically reduced representations are not always guaranteed to exist. For example, in Figure 4b, it is easy to verify that the condition $I_{\mu }\left(R_{\mathrm{ex}};S=□\right)<I_{\mu }\left(R_{\mathrm{su}};S=□\right)$ holds for any μ(◯)≠0. Therefore, no stochastic mapping can transform $R_{\mathrm{ex}}$ into $R_{\mathrm{su}}$ in such a way that $P_{\mathrm{ex}}\left(r|s\right)$ is converted into $P_{\mathrm{su}}\left(r|s\right)$. Schneidman et al. [9] employed an analogous example, but involving different neurons instead of response aspects. The two examples of Figure 4 motivate the following theorem:

**Theorem** **1.** *No deterministic mapping $R_{\mathrm{ex}}\to R_{\mathrm{su}}$ exists transforming the conditional probability $P_{\mathrm{ex}}\left(r|s\right)$ into its noise-independent version $P_{\mathrm{su}}=P_{NI}\left(r|s\right)$ defined in Equation (17). Stochastic mappings $R_{\mathrm{ex}}\to R_{\mathrm{su}}$ may or may not exist, depending on the conditional probability $P_{\mathrm{ex}}\left(r|s\right)$*.

**Proof.**  See Appendix B.2. □

**Figure 5:**
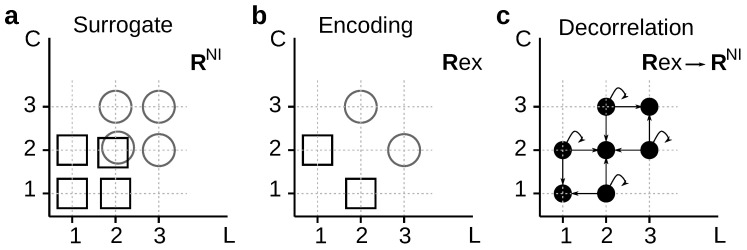
Stochastically reduced representations that ignore noise correlations may depend on them.(**a**) Cartesian coordinates representing a hypothetical experiment in which two different stimuli, □ and ◯, elicit single neuron responses ($R_{\mathrm{su}}=R_{NI}$) that are completely characterized by their first-spike latency (*L*) and spike counts (*C*). Both *L* and *C* are noise independent; (**b**) Cartesian coordinates representing a hypothetical experiment with the same marginal probabilities $P_{\mathrm{ex}}\left(l|s\right)$ and $P_{\mathrm{ex}}\left(c|s\right)$ as in panel (a), with one among many possible types of noise correlations between *L* and *C*; (**c**) Stimulus-independent stochastic function transforming the noise-correlated responses $R_{\mathrm{ex}}$ of panel (b) into the noise-independent responses $R_{\mathrm{su}}=R_{NI}$ of panel (a). The transition probabilities $Q(r_{\mathrm{su}}|r_{\mathrm{ex}})$ are given in Equation (20), and they bear an explicit dependence on the amount of noise correlations.

In addition, when a stochastic mapping $R_{\mathrm{ex}}\to R_{\mathrm{su}}$ exists, the values of the probabilities $Q(r_{\mathrm{su}}|r_{\mathrm{ex}})$ may well depend on the discarded response aspect, as well as on the preserved response aspects. We mention this fact, because when assessing the relevance of noise correlations, the marginals $P_{\mathrm{ex}}\left(r_{i}|s\right)$ suffice for us to write down the surrogate distribution $P_{\mathrm{su}}\left(r|s\right)=P_{NI}\left(r|s\right)$, with no need to know the full distribution $P_{\mathrm{ex}}\left(r|s\right)$ containing the noise correlations. One could have hoped that perhaps also the mapping $R_{\mathrm{ex}}\to R_{\mathrm{su}}$ (assuming that such a mapping exists) could be calculated with no knowledge of the noise correlations. This is, however, not always true, as stated in the theorem below. Two experiments with the same marginals and different amounts of noise correlations may require different mappings to eliminate noise correlations, as illustrated in the the example of Figure 5. More formally:

**Theorem** **2.** *The transition probabilities $Q(r_{\mathrm{su}}|r_{\mathrm{ex}})$ of stochastic codes that ignore noise correlations may depend both on the marginal likelihoods (preserved at the output of the mapping), and on the noise correlations (eliminated at the output of the mapping)*.

**Proof.**  See Appendix B.3. □

The solution of Equation (10) for the example of Figure 5 is

(20) $$Q\left(r_{\mathrm{su}}|r_{\mathrm{ex}}\right)=\frac{1}{2}\left[\begin{array}{ccccccc} \bar{a} & 2a & 0 & \bar{a} & 0 & 0 & 0\\ a & 0 & 2\bar{a} & a & 0 & 0 & 0\\ 0 & 0 & 0 & b & 2\bar{b} & 0 & b\\ 0 & 0 & 0 & \bar{b} & 0 & 2b & \bar{b} \end{array}\right],$$

where each response is defined by a vector [L,C], and rows and columns enumerate the elements of the ordered sets $R_{ex}=\left\{\left[1,2\right],\left[2,1\right],\left[2,3\right],\left[3,2\right]\right\}$ and $R_{su}=\left\{\left[1,1\right],\left[1,2\right],\left[2,1\right],\left[2,2\right],\left[2,3\right],\left[3,2\right],\left[3,3\right]\right\}$ from where $R_{\mathrm{ex}}$ and $R_{\mathrm{su}}$ are sampled, respectively. In Equation (20), $a=P(R_{\mathrm{ex}}=\left[1,2\right]|S=□)$; and $b=P(R_{\mathrm{ex}}=\left[3,2\right]|S=◯)$. The fact that the matrix in Equation (20) bears an explicit dependence on these parameters–and not only on $P_{\mathrm{ex}}\left(L|S\right)$ and $P_{\mathrm{ex}}\left(C|S\right)$–implies that the transformation between $R_{\mathrm{ex}}$ and $R_{\mathrm{su}}$ depends on the amount of noise correlations in $R_{\mathrm{ex}}$.

### 2.3. Multiple Measures to Assess the Relevance of a Specific Response Feature

The importance of a specific response feature has been previously quantified in many ways (see [17,30] and references therein), which have oftentimes led to heated debates about their merits and drawbacks [9,11,12,17,31,32,33]. Here we consider several measures, to underscore the diversity of the meanings with which the relevance of a given feature has been assessed so far. They are mathematically defined as

(21) $$\begin{array}{c} \Delta I_{R_{\mathrm{su}}}=I_{R_{\mathrm{ex}}}-I_{R_{\mathrm{su}}} \end{array}$$

(22) $$\begin{array}{c} \Delta I_{\hat{S}}=I_{R_{\mathrm{ex}}}-I_{\hat{S}}^{\beta } \end{array}$$

(23) $$\begin{array}{c} \Delta I_{\hat{S}}=I_{R_{\mathrm{ex}}}-I_{\hat{S}}^{\beta } \end{array}$$

(24) $$\begin{array}{c} \Delta A_{R_{\mathrm{su}}}=A_{R_{\mathrm{ex}}}^{R_{\mathrm{ex}}}-A_{R_{\mathrm{su}}}^{R_{\mathrm{su}}} \end{array}$$

(25) $$\begin{array}{c} \Delta I_{}^{D}=\sum_{s,r}P_{\mathrm{ex}}\left(s,r\right)\ln\frac{P_{\mathrm{ex}}\left(s|r\right)}{P_{\mathrm{su}}\left(s|r\right)} \end{array}$$

(26) $$\begin{array}{c} \Delta I_{}^{DL}=\min_{\theta }\sum_{s,r}P_{\mathrm{ex}}\left(s,r\right)\ln\frac{P_{\mathrm{ex}}\left(s|r\right)}{P_{\mathrm{su}}\left(s|r,\theta \right)} \end{array}$$

(27) $$\begin{array}{c} \Delta I_{}^{LS}=I_{R_{\mathrm{ex}}}-I_{\hat{S}}^{\alpha } \end{array}$$

(28) $$\begin{array}{c} \Delta I_{}^{B}=I_{R_{\mathrm{ex}}}-I_{\hat{S}}^{\alpha } \end{array}$$

(29) $$\begin{array}{c} \Delta A_{}^{B}=A_{R_{\mathrm{ex}}}^{R_{\mathrm{ex}}}-A_{R_{\mathrm{ex}}}^{R_{\mathrm{su}}} \end{array}$$

**Figure 6:**
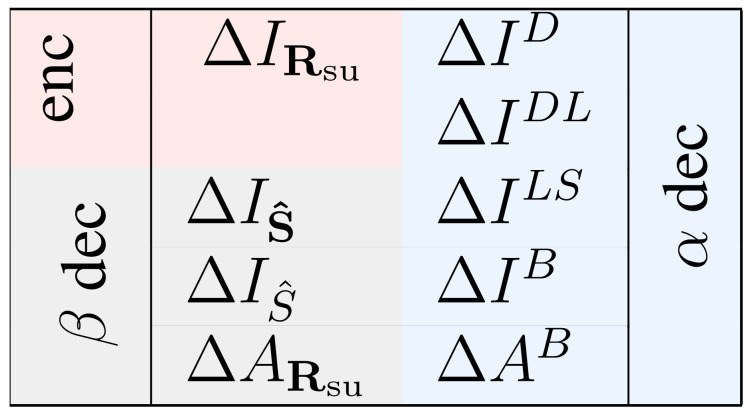
Relations between the measures defined in Equations (21)–(29). The four measures on the left are either encoding-oriented ($\Delta I_{R_{\mathrm{su}}}$, on a pink background), or half-way between encoding- and decoding-oriented (the last three, gray background). The five measures on the right are all decoding-oriented (light-blue background). Each measure on the left has a conceptually related measure on the right on the same line, except for $\Delta I_{R_{\mathrm{su}}}$, which has two associated decoding-oriented measures: $\Delta I^{D}$ and $\Delta I^{LD}$. The distinction between the measures on pink and on gray background relies on the fact that $\Delta I_{R_{\mathrm{su}}}$ does not involve a decoding process. Instead, $\Delta I_{\hat{S}},\;\Delta I_{\hat{S}}$ and $\Delta A_{R_{\mathrm{su}}}$ decode a stimulus (or rank the stimuli) with decoding method β. This decoding is not meant to be applicable to real experiments, since (as opposed to the truly decoding-oriented measures on the right, that operate with method α) the decoding is applied to the surrogate responses $R_{\mathrm{su}}$, not the real ones $R_{\mathrm{ex}}$.

Equations (22)–(24) are based on matched decoders, that is, decoders operating on responses governed by the same probability distribution involved in their construction (method β). Instead, Equations (25)–(28) are based on the operation of mismatched decoders (method α). Each measure of Equations (21)–(24) has one or two homologous measures in Equations (25)–(29), as illustrated in Figure 6.

We here describe the measures briefly, and refer the interested reader to the original papers.

In Equation (21), $I_{R_{\mathrm{ex}}}$ and $I_{R_{\mathrm{su}}}$ are the mutual informations between the set of stimuli and a set of responses governed by the distributions $P_{\mathrm{ex}}\left(r|s\right)$ and $P_{\mathrm{su}}\left(r|s\right)$, respectively. Thus, $\Delta I_{R_{\mathrm{su}}}$ is the simplest way in which the information encoded by the true responses can be compared with that of the surrogate responses. This comparison has been employed for more than six decades in neuroscience [34,35] to study, for example, the encoding of different stimulus features in spike counts, in synchronous spikes, and in other forms of spike patterns, both in single neurons and populations (see [30] and references therein).

The measure $\Delta I_{}^{D}$ defined in Equation (25) was introduced by Nirenberg et al. [8] to study the role of noise correlations, and was later extended to arbitrary deterministic mappings [10,12,13]. Here we use the supra-script *D* to indicate that the measure is the “divergence” (in the Kullback-Leibler sense) between the posterior stimulus distributions calculated with the real and the surrogate responses, respectively. In [10], Nirenberg and Latham argued that the important feature of $\Delta I_{}^{D}$ is that it represents the information loss of a mismatched decoder trained with $P_{\mathrm{su}}\left(r|s\right)$ but operated on the real responses, sampled from $P_{\mathrm{ex}}\left(r|s\right)$. Not before long, Schneidman et al. [9] noticed that $\Delta I_{}^{D}$ can exceed $I_{R_{\mathrm{ex}}}$. The interpretation of $\Delta I_{}^{D}$ as a measure of information loss would imply that decoders trained with surrogate responses can lose more information than the one encoded by the real response. In fact, $\Delta I_{}^{D}$ tends to infinity if $P_{\mathrm{su}}\left(s|r\right)\to 0$ when P(s|r)>0 for some *s*. In the limit, $\Delta I_{}^{D}$ becomes undefined when $P_{\mathrm{su}}\left(r\right)=0$ and $P_{\mathrm{ex}}\left(r\right)>0$. To avoid this peculiar behavior, Latham and Nirenberg generalized the theoretical framework used to derive $\Delta I^{D}$ [11], giving rise to the measure $\Delta I_{}^{DL}$ of Equation (26). Here, the supra-script DL makes reference to “Divergence Lowest”, since the measure was presented as the lowest possible information loss of a decoder trained with $P_{\mathrm{su}}\left(r|s\right)$. In the definition of $\Delta I^{LD}$, the parameter θ is a real scalar. The distribution $P_{\mathrm{su}}\left(s|r,\theta \right)$ was defined by Latham and Nirenberg [11] as proportional to $P\left(s\right)P_{\mathrm{su}}{\left(r|s\right)}^{\theta }$. This definition has several problems, as discussed in [11,17,36,37,38,39]. In Appendix B.1 we demonstrate a theorem that resolves the issues appearing in previous definitions, and justifies the use of

(30) $$P_{\mathrm{su}}\left(s|r,\theta \right)\propto \left\{\begin{array}{cc} P(s) & \mathrm{if}\;\exists \hat{s},\;\hat{r}\mathrm{such}\;\mathrm{that}\;P_{\mathrm{ex}}\left(\hat{r}|\hat{s}\right)>P_{\mathrm{su}}\left(\hat{r}|\hat{s}\right)=0\\ 0 & \mathrm{if}\;P_{\mathrm{su}}\left(r|s\right)=P_{\mathrm{ex}}\left(r|s\right)=0\;\mathrm{for}\;\mathrm{some}\;\mathrm{but}\;\mathrm{not}\;\mathrm{all}\;s\\ P\left(s\right)P_{\mathrm{su}}{\left(r|s\right)}^{\theta } & \mathrm{otherwise} \end{array}\right.$$

From the conceptual point of view, $\Delta I^{DL}$ represents the information loss of a mismatched decoder trained with $P_{\mathrm{su}}\left(r|s\right)$ and operated on $R_{\mathrm{ex}}$. Latham and Nirenberg [11] showed that, unlike $\Delta I_{}^{D}$, it is possible to demonstrate that $\Delta I_{}^{DL}\leq I_{R_{\mathrm{ex}}}$. Hence, $\Delta I_{}^{DL}$ never yields a tested feature encoding more information than the full response. The proof in [11] ignored a few specific cases that we discuss in the Theorem A1 of Appendix B.1. Still, even in those additional cases, the inequality $\Delta I_{}^{DL}\leq I_{R_{\mathrm{ex}}}$ holds.

In Equations (22) and (23), $\hat{S}$ and $\hat{S}$ denote a sorted stimulus list and the most-likely stimulus, respectively, both decoded by evaluating Equation (6) (or its ranked version) on a response r sampled from the surrogate distribution $P_{\mathrm{su}}\left(r|s\right)$ (method β). Estimating mutual informations using decoders can be traced back at least to Gochin et al. [40], and comparing the estimations of two decoders that take different response features into account, at least to Warland et al. [41].

The measures $\Delta I_{\hat{S}}$ and $\Delta I_{\hat{S}}$ are paired with $\Delta I_{}^{LS}$ and $\Delta I_{}^{B}$, respectively, since the latter are obtained from the former when replacing the decoding method from β to α. The measure $\Delta I_{}^{LS}$ was introduced by Ince et al. [20], and quantifies the difference between the information in $R_{\mathrm{ex}}$, and the one in the output of decoders that, after observing a variable r sampled with distribution $P_{\mathrm{ex}}\left(r|s\right)$ (method α), produce a stimulus list sorted according to $P_{\mathrm{su}}\left(s|r\right)$. The supra-script LS indicates “List of Stimuli”. Similarly, $\Delta I_{}^{B}$, quantifies the difference between the information encoded in $R_{\mathrm{ex}}$ and that encoded in the output of a decoder trained by inserting $P_{\mathrm{su}}\left(s|r\right)$ into Equation (6), and operated on r sampled with distribution $P_{\mathrm{ex}}\left(r|s\right)$ (method α). The supra-script *B* stands for the “Bayesian” nature of the involved decoder. The use of these measures can be traced back at least to Nirenberg et al. [8], although in that case, decoders were restricted to be linear. The measure $\Delta I_{\hat{S}}$ of Equation (22) is new, and we have introduced it here as the homologous of $\Delta I_{}^{LS}$. When the number of stimuli is two, $\Delta I_{\hat{S}}=\Delta I_{\hat{S}}$, since selecting the optimal stimulus is (as a computation) in one-to-one correspondence with ranking the two candidate stimuli.

The accuracy loss $\Delta A_{R_{\mathrm{su}}}$ defined in Equation (24) entails the comparison between the performance of two decoders, one trained with and applied on $R_{\mathrm{ex}}$, and one trained with and applied on $R_{\mathrm{su}}$. Such comparisons have also a long history in neuroscience [42,43] (see [9,12] for further discussion). The accuracy loss $\Delta A^{B}$ also compares two decoders. The first, is the same as for $\Delta A_{R_{\mathrm{su}}}$, but the second is trained with $R_{\mathrm{su}}$ and applied on $R_{\mathrm{ex}}$.

The measures $\Delta I_{}^{LS}$, $\Delta I_{}^{B}$, and $\Delta A_{}^{B}$ are undefined if the actual responses $R_{\mathrm{ex}}$ are not contained in the set of surrogate responses $R_{\mathrm{su}}$. In other words, a decoder constructed with $P_{\mathrm{su}}\left(r|s\right)$ does not know what output to produce when evaluated in a response r for which $P_{\mathrm{su}}\left(r\right)=0$. This situation never happens when evaluating the relevance of noise correlations with $P_{\mathrm{su}}=P_{NI}$, but it may well be encountered in more general situations, as for example, in Figure 3B.

### 2.4. Relating the Values Obtained with Different Measures

If a mapping $R_{\mathrm{ex}}\to R_{\mathrm{su}}$ exists transforming $P_{\mathrm{ex}}\left(r|s\right)$ into $P_{\mathrm{su}}\left(r|s\right)$, we may use the decoding procedure of Equation (6) to construct the transformation chain $R_{\mathrm{ex}}\to R_{\mathrm{su}}\to \hat{S}\to \hat{S}$ [17,44]. Consequently, $\Delta I_{R_{\mathrm{su}}}$, $\Delta I_{\hat{S}}$ and $\Delta I_{\hat{S}}$ can be interpreted as accumulated information losses after the first, second and third transformations, respectively, and $\Delta A_{R_{\mathrm{su}}}$, as the accuracy loss after the first transformation. The data processing theorems (Section 2.1.3) ensure that these measures are never negative. This property, however, cannot be guaranteed in the absence of a reduced transformation $R_{\mathrm{ex}}\to R_{\mathrm{su}}$, stochastic or deterministic. Indeed, in the example of Figure 4b, if both stimuli are equiprobable, and both responses $R_{\mathrm{ex}}$ associated with ◯ are equiprobable, then $\Delta I_{R_{\mathrm{su}}}=\Delta I_{\hat{S}}=\Delta I\hat{S}\approx -79\%$ of $I_{R_{\mathrm{ex}}}\approx 0.31\;\mathrm{bits}$, implying that the surrogate responses encode more information about the stimulus than the original, experimental responses. Removing the correlations between spike count and latency, hence, increases the information, so correlations can be concluded to be detrimental to information encoding.

Irrespective of whether a (deterministic or stochastic) mapping $R_{\mathrm{ex}}\to R_{\mathrm{su}}$ exists, the data processing inequality guarantees that $\Delta I_{R_{\mathrm{su}}}\leq \Delta I_{\hat{S}}\leq \Delta I_{\hat{S}}$, since $\hat{S}$ is a deterministic function of $R_{\mathrm{su}}$, and $\hat{S}$ is a deterministic function of $\hat{S}$. The inequality holds irrespective of the sign of each measure.

All decoder-oriented measured are guaranteed to be non-negative. The very definitions of $\Delta I_{}^{D}$ and of $\Delta I_{}^{DL}$ imply they cannot be negative, since they are both Kullback-Leibler divergences between two probability distributions. The sequence of reduced transformations $R_{\mathrm{ex}}\to \hat{S}\to S$, in turn, guarantees the non-negativity of $\Delta I_{}^{LS},\;\Delta I_{}^{B}$ and $\Delta A_{}^{B}$, through the Data Processing Inequalities.

**Figure 7:**
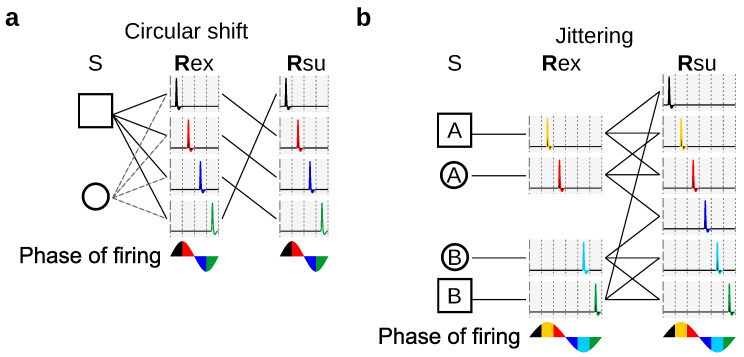
Stochastic codes may play different roles in encoding and decoding. (**a**) Hypothetical experiment with two stimuli □ and ◯, which are transformed (solid and dashed lines) into neural responses containing a single spike (C=1) fired at different phases (Φ) with respect to a cycle of 20 ms period starting at stimulus onset. The phases have been discretized in intervals of size π/2 and wrapped to the interval [0,2π). The encoding process is followed by a circular phase-shift that transforms $R_{\mathrm{ex}}=\Phi$ into another code $R_{\mathrm{su}}=\hat{\Phi }$ with transition probabilities $Q(r_{\mathrm{su}}|r_{\mathrm{ex}})$ defined by Equation (31). The set of all $R_{\mathrm{su}}$ coincides with the set of all $R_{\mathrm{ex}}$; (**b**) Same as (a), except that stimuli are four (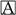, Ⓐ, 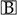, and Ⓑ), and phases are measured with respect to a cycle of 30ms period and discretized in intervals of size π/3. The encoding process is followed by a stochastic transformation (lines on the right) that introduces jitter, thereby transforming $R_{\mathrm{ex}}=\Phi$ into another code $R_{\mathrm{su}}=\hat{\Phi }$ with transition probabilities $Q(r_{\mathrm{su}}|r_{\mathrm{ex}})$ defined by Equation (32).

In order to assess whether decoding-oriented measures are always larger or smaller than their encoding (or gray) counterparts, we performed a numerical exploration comparing each encoding/gray-oriented measure with its decoding-oriented homologue. The exploration was conducted by calculating the values of these measures for a large collection of possible stimulus prior probabilities P(s), and response conditional probabilities $P_{\mathrm{ex}}\left(r|s\right)$ in the examples of Figure 2, Figure 3, Figure 4 and Figure 7. The details of the numerical exploration are in Appendix A. The measures in the first group were sometimes greater and sometimes smaller than those of the second group, depending on the case and the probabilities (Table 1). Consequently, our results demonstrate that there is no general rule by which measures of one type bound the measures of the other type.

The exploration also included the example of Figure 7a. In panel (a), the transition probabilities are

(31) $$Q\left(r_{\mathrm{su}}|r_{\mathrm{ex}}\right)=\left[\begin{array}{cccc} 0 & 1 & 0 & 0\\ 0 & 0 & 1 & 0\\ 0 & 0 & 0 & 1\\ 1 & 0 & 0 & 0 \end{array}\right],$$

where rows and columns enumerate the elements of the ordered sets $R_{ex}=R_{su}=\left\{\left[1\right],\left[2\right],\left[3\right],\left[4\right]\right\}$ from where both $R_{\mathrm{ex}}$ and $R_{\mathrm{su}}$ are sampled. For panel b,

(32) $$Q\left(r_{\mathrm{su}}|r_{\mathrm{ex}}\right)=\frac{1}{2}\left[\begin{array}{cccccc} 2\bar{a} & a & a & 0 & 0 & 0\\ 0 & b & b & 2\bar{b} & 0 & 0\\ 0 & 0 & 0 & 2\bar{b} & b & b\\ 2\bar{a} & 0 & 0 & 0 & a & a \end{array}\right],$$

with 0<a,b<1, rows enumerating the elements of $R_{ex}=\left\{\left[2\right],\left[3\right],\left[5\right],\left[6\right]\right\}$, and columns those of $R_{su}=\left\{\left[1\right],\left[2\right],\left[3\right],\left[4\right],\left[5\right],\left[6\right]\right\}$.

An important issue is to identify the situations in which $\Delta I_{R_{\mathrm{su}}}$ gives exactly the same result as either $\Delta I_{}^{D}$ or $\Delta I_{}^{DL}$. It is not easy to determine the conditions for the equality between $\Delta I_{R_{\mathrm{su}}}$ and $\Delta I_{}^{DL}$. Yet, for the equality between $\Delta I_{R_{\mathrm{su}}}$ and $\Delta I_{}^{D}$, and in the specific case in which $P_{\mathrm{su}}\left(r|s\right)=P_{NI}\left(r|s\right)$ as given by Equation (17), the following theorem holds.

**Theorem** **3.** *When assessing the relevance of noise correlations, $\Delta I_{}^{D}=\Delta I_{R_{\mathrm{su}}}$ if and only if*

(33) $$\lambda =\sum_{r}\left[P_{\mathrm{ex}}\left(r\right)-P_{\mathrm{su}}\left(r\right)\right]\log_{2}\left[P_{\mathrm{su}}\left(r\right)\right]=0.$$

*Moreover, λ≶0 implies that $\Delta I_{}^{D}≶\Delta I_{R_{\mathrm{su}}}$*.

**Proof.**  See Appendix B.4. □

Equation (33) implies that neither the prior stimulus probabilities P(s) nor the conditional response probabilities $P_{\mathrm{ex}}\left(r|s\right)$ intervene in the condition for the equality, beyond the effect they have in fixing the value of $P_{\mathrm{ex}}\left(r\right)$ and $P_{\mathrm{su}}\left(r\right)$. Each response r makes a contribution to the value of λ, which favours $\Delta I^{D}$ whenever $P_{\mathrm{su}}\left(r\right)>P_{\mathrm{ex}}\left(r\right)$, and $I_{R_{\mathrm{ex}}}$ in the opposite case. As pointed out by [10], all responses r for which $P_{\mathrm{ex}}\left(r\right)=0$ and $P_{\mathrm{su}}\left(r\right)>0$ give a null contribution to $\Delta I^{D}$, and a negative contribution to $I_{R_{\mathrm{ex}}}$, implying that correlations in such responses are irrelevant for decoding, and detrimental to encoding.

The fact that encoding-oriented measures neither bound nor are bounded by decoding-oriented measures is a daunting result. If, when working in a specific example, one gets a positive value with one measure and a negative value with another, the interpretation must carefully distinguish between the two paradigms. One may wonder, however, if such distinction is also required when correlations are absolutely essential for one of the measures, in that they capture the whole of the encoded information. Could the other measure conclude that they are irrelevant? Or that they are only mildly relevant? Luckily, in this case, the answer is negative. In other words, when the tested feature is fundamental, then $\Delta I_{}^{D}$ and $\Delta I_{R_{\mathrm{su}}}$ coincide, and no conflict arises between encoding and decoding, as proven by the following theorem:

**Theorem** **4.** *$\Delta I_{}^{DL}=I_{R_{\mathrm{ex}}}$ if and only if $\Delta I_{R_{\mathrm{su}}}=I_{R_{\mathrm{ex}}}$, regardless of whether stochastic codes exist that map the actual responses $R_{\mathrm{ex}}$ into the surrogate responses $R_{\mathrm{su}}=R_{NI}$ generated assuming noise independence*.

**Proof.**  See Appendix B.5. □

The conclusion is that if a given feature is 100% relevant for encoding, then it is also 100% relevant for decoding, and vice versa. Hence, although $\Delta I_{R_{\mathrm{su}}}$ and $\Delta I_{}^{DL}$ often differ in the relevance they ascribe to a given feature, the discrepancy is only encountered when the tested feature is not the only informative feature in play. When the removal of the feature is catastrophic (in the sense that it brings about a complete information loss), then both $\Delta I_{R_{\mathrm{su}}}$ and $\Delta I_{}^{DL}$ diagnose the situation equally.

### 2.5. Relation between Measures Based on Decoding Strategies α and β

The results of Table 1 may seem puzzling because decoding happens after encoding. Therefore—one may naively reason—the data processing theorems should have forbidden both $\Delta I_{R_{\mathrm{su}}}$ to surpass $\Delta I_{}^{D}$, $\Delta I_{}^{DL}$, or $\Delta I_{}^{B}$, as well as $\Delta A_{R_{\mathrm{su}}}$ to surpass $\Delta A_{}^{B}$. However, even though decoding indeed happens after encoding, the data processing theorem is not violated. The theorem certainly ensures that $\Delta I_{R_{\mathrm{su}}}$ and $\Delta A_{R_{\mathrm{su}}}$ constitute lower bounds for measures related to decoders that operate on responses generated by $P_{\mathrm{su}}\left(r|s\right)$, but not for measures related to decoders that operate on responses generated by $P_{\mathrm{ex}}\left(r|s\right)$, such as happens with $\Delta I_{}^{D}$, $\Delta I_{}^{DL}$, $\Delta I_{}^{B}$, and $\Delta A_{}^{B}$.

This observation about the validity of the data processing inequality is different from the one discussed in Section 2.2. There, we discussed the conditions under which $\Delta I_{R_{\mathrm{su}}}$ could be guaranteed to be non-negative, the crucial factor being the existence of a stochastic mapping $R_{\mathrm{ex}}\to R_{\mathrm{su}}$. Now we are discussing a different aspect, regarding whether decoding-related measures can or cannot be bounded by encoding-oriented measures. The conclusion is that in general terms, the answer is negative, because decoding-related measures operate with decoding strategy α, a strategy never addressed by the encoding measures. The surrogate variable $R_{\mathrm{su}}$ participating in the encoding measure $\Delta I_{R_{\mathrm{su}}}$ is *not* the response decoded by the measures of Equations (25)–(28), so the data processing inequalities need not hold. That being said, there are specific instances in which both types of measures coincide, two of them discussed in Theorems 3 and 4 and a third case later in Theorem 5.

Other explanations have been given in the literature for the fact that sometimes, decoding oriented measures surpass their encoding counterparts. For example, it has been alleged [10] that when $\Delta I_{}^{D},\;\Delta I_{}^{DL}$ or $\Delta I_{}^{B}$ are smaller than $\Delta I_{R_{\mathrm{su}}}$, this is either due to (a) the impossibility to define a stimulus-independent reduction $R_{\mathrm{ex}}\to R_{\mathrm{su}}$ that yields $P_{\mathrm{ex}}\left(r|s\right)\to P_{\mathrm{su}}\left(r|s\right)$ (and therefore the data-processing inequality is not guaranteed to hold), or due to (b) the fact that surrogate responses often sample values of response space that are never reached by real responses (and therefore, the losses of matched decoders may be larger than the ones of mismatched ones). However, Figure 2c constitutes a counterexample of both arguments, since there, the stimulus-independent stochastic reduction exists, and the response set of $R_{\mathrm{ex}}$ and $R_{\mathrm{su}}$ coincide.

One could also wonder whether the discrepancy between the values obtained with encoding-oriented measures and decoding-oriented measures only occurs in examples where a stochastic reduction $R_{\mathrm{ex}}\to R_{\mathrm{su}}$ exists, and the involved transition matrix $Q(r_{\mathrm{su}}|r_{\mathrm{ex}})$ depends on the joint probabilities $P_{\mathrm{ex}}\left(r,s\right)$, and not only on the marginals, as discussed in Theorem 2. However, Figure 2b,c provide examples in which $Q(r_{\mathrm{su}}|r_{\mathrm{ex}})$ does not depend on P(r,s), and yet, the discrepancies are still observed.

The distinction between decoding strategies α and β is also crucial when using the measure $\Delta I_{}^{D}$. This measure was introduced by Nirenberg et al. [8] for the specific case in which the tested feature is the amount of noise correlations, that is, when $P_{\mathrm{su}}\left(s|r\right)=P_{NI}\left(s|r\right)$. The measure was later extended to arbitrary deterministic mappings $R_{\mathrm{su}}=f\left(R_{\mathrm{ex}}\right)$ [10,12,13], with the instruction to use an expression like Equation (25), but with $P_{\mathrm{su}}\left(s|r\right)$ replaced by $P\left(s|R_{\mathrm{su}}=f\left(r\right)\right)=P_{\mathrm{su}}\left(s|f\left(r\right)\right)$. It should be noted, however, that as soon as this replacement is made, $\Delta I_{}^{D}$ becomes exactly equal to $\Delta I_{R_{\mathrm{su}}}$. Specifically, the measure $\Delta I_{}^{D}$ now describes the information loss of a decoder that operates on a response variable generated with the surrogate distribution $P_{\mathrm{su}}\left(r|s\right)$ (decoding method β). If we want to keep the original spirit, and associate $\Delta I_{}^{D}$ with a decoder that operates on a response variable generated with the real distribution $P_{\mathrm{ex}}\left(r|s\right)$ (decoding method α), in Equation (25), $P_{\mathrm{su}}\left(s|r\right)$ should not be modified. Only the evaluation of the surrogate variable $R_{\mathrm{su}}$ in the experimentally observed value $R_{\mathrm{ex}}=r$ describes a mismatched decoder constructed with $P_{\mathrm{su}}\left(r|s\right)$ and operated on $R_{\mathrm{ex}}$ (mathematical details in Appendix C).

### 2.6. Assessing the Type of Information Encoded by Individual Response Features

When the stimulus contains several attributes (as shape, color, sound, etc.), by removing a specific response feature it is possible to assess not only *how much* information is encoded by the feature, but also, *what type* of information. Identifiying the type of encoded information implies determining the stimulus feature represented by the tested response feature. As shown in this section, the type of encoded information is as dependent on the method of removal as is the amount. In other words, the different measures defined in Equations (21)–(29) sometimes associate a feature with the encoding of different stimulus attributes.

**Figure 8:**
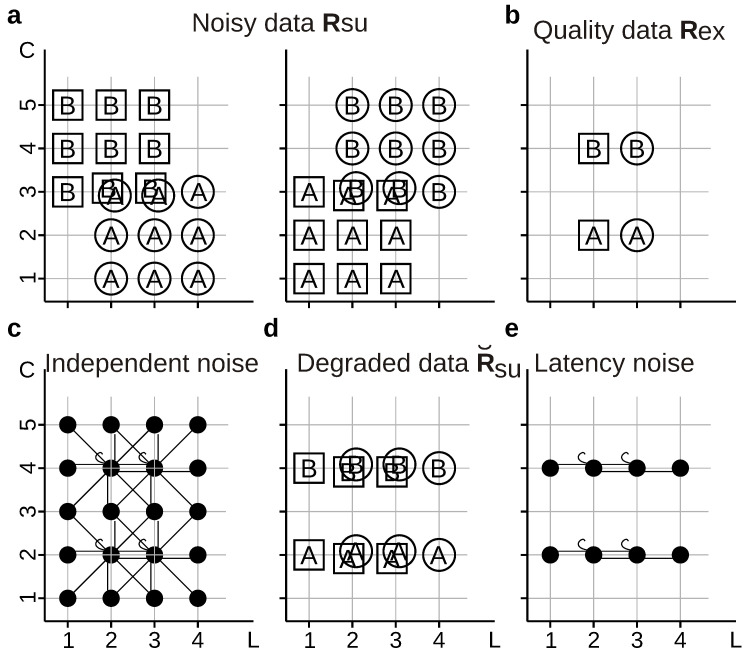
Assessing the amount and type of information encoded by. (**a**) Noisy data $R_{\mathrm{su}}=\left[L,C\right]$ recorded in response of the compound stimulus $S=[S_{F},S_{L}]$; (**b**) Quality data $R_{\mathrm{ex}}=\left[L,C\right]$ recorded in the case of panel (a), but without noise; (**c**) Stimulus-independent stochastic transformation with transition probabilities $Q(r_{\mathrm{su}}|r_{\mathrm{ex}})$ given by Equation (34), that introduces independent noise both in the latencies and in the spike counts, thereby transforming $R_{\mathrm{ex}}$ into $R_{\mathrm{su}}$ and rendering $R_{\mathrm{su}}$ a stochastic code; (**d**) Degraded data $\overset{˘}{R}$ obtained by adding latency noise to the quality data; (**e**) Representation of the stimulus-independent stochastic transformation $R_{\mathrm{ex}}\to \overset{˘}{R}$ with transition probabilities $Q(\overset{˘}{r}|r_{\mathrm{ex}})$ given by Equation (35) that adds latency noise in panel (d).

In the example of Figure 8, we use four compound stimuli $S=[S_{F},\;S_{L}]$, generated by choosing independently a frame ($S_{F}=□$ or ◯) and a letter ($S_{L}=A$ or B), thereby yielding 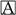, Ⓐ, 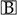, and Ⓑ. Stimuli are transformed into neural responses R=[L,C] with different number of spikes (1≤C≤5) fired at different first-spike latencies (1≤L≤4; time has been discretized in 5 ms bins). Latencies are only sensitive to frames whereas spikes counts are only sensitive to letters, thereby constituting independent-information streams: $P\left(s,r\right)=P\left(s_{F},l\right)P\left(s_{L},c\right)$ [33]. The equality in the numerical value of two measures does not imply that both measures assign the same meaning to the information encoded by the tested response feature. Indeed, the two measures may sometimes report the tested response feature to encode two different aspects of the set of stimuli. Consider a decoder that is trained using the noisy data $R_{\mathrm{su}}$ shown in Figure 8a, but it is asked to operate on either the same noisy data with which it was trained (strategy β), or with the quality data $R_{\mathrm{ex}}$ of Figure 8b (strategy α). The information losses $\Delta I_{R_{\mathrm{su}}}$, $\Delta I_{}^{D}$, and $\Delta I_{}^{DL}$ are all equal to 50% of $I(S,R_{\mathrm{ex}})=2\;\mathrm{bits}$. Therefore, the information loss is independent of whether, in the operation phase, the decoder is fed with responses generated with $P_{\mathrm{su}}\left(r|s\right)$ or with $P_{\mathrm{ex}}\left(r|s\right)$.

The transformation $Q(r_{\mathrm{su}}|r_{\mathrm{ex}})$ causes some responses $R_{\mathrm{su}}$ to occur for all stimuli, so when decoding with method β, some information about frames is lost (that is, $I(S_{F},R_{\mathrm{su}})\approx 33\%$ of $I(S_{F},R_{\mathrm{ex}})=1\;\mathrm{bit}$), as well as some information about letters (that is, $I(S_{L},R_{\mathrm{su}})\approx 67\%$ of $I(S_{L},R_{\mathrm{ex}})=1\;\mathrm{bit}$). In other words, decoding $R_{\mathrm{su}}$ causes a partial information loss $\Delta I_{R_{\mathrm{su}}}$ that is composed of both frame and letter information. Instead, when decoding $R_{\mathrm{ex}}$ with method α, there is no information loss about letters: For the responses $R_{\mathrm{ex}}$ that actually occur, the decoder trained with $R_{\mathrm{su}}$ can perfectly identify the letters, because $P_{\mathrm{su}}\left(C=2|S_{L}=A\right)=P_{\mathrm{su}}\left(C=4|S_{L}=B\right)=1$. The information about frames, on the other hand, is completely lost, since $P_{\mathrm{su}}\left(l|□\right)=P_{\mathrm{su}}\left(l|◯\right)$ whenever *l* adopts a value that actually occurs in $R_{\mathrm{ex}}$, namely 2 or 3. This example shows that the fact that two decoding procedures give the same numerical loss does not mean that they draw the same conclusions regarding the role of the tested feature in the neural code. Ananalogous computations yield analogous results for the hypothetical experiment shown in Figure 7b.

If responses $r_{\mathrm{ex}}$ and $r_{\mathrm{su}}$ are written as vectors [L,C], and the values of $Q(r_{\mathrm{su}}|r_{\mathrm{ex}})$ are arranged in a rectangular structure, in Figure 8c the transition probabilities are

(34) $$Q\left(r_{\mathrm{su}}|r_{\mathrm{ex}}\right)=\frac{1}{9}\left[\begin{array}{cccccccccccccccccccc} 1 & 1 & 1 & 0 & 1 & 1 & 1 & 0 & 1 & 1 & 1 & 0 & 0 & 0 & 0 & 0 & 0 & 0 & 0 & 0\\ 0 & 1 & 1 & 1 & 0 & 1 & 1 & 1 & 0 & 1 & 1 & 1 & 0 & 0 & 0 & 0 & 0 & 0 & 0 & 0\\ 0 & 0 & 0 & 0 & 0 & 0 & 0 & 0 & 1 & 1 & 1 & 0 & 1 & 1 & 1 & 0 & 1 & 1 & 1 & 0\\ 0 & 0 & 0 & 0 & 0 & 0 & 0 & 0 & 0 & 1 & 1 & 1 & 0 & 1 & 1 & 1 & 0 & 1 & 1 & 1 \end{array}\right],$$

where rows and columns indicate the ordered sets $R_{ex}=\left\{\left[2,2\right],\left[3,2\right],\left[2,4\right],\left[3,4\right]\right\}$ and $R_{su}=\left\{1,2,3,4\right\}\times \left\{1,2,3,4,5\right\}$, where × denotes the Cartesian product with colexicographical order, that is, ordered as [1,1],[2,1],[3,1],[4,1],[1,2], etc. In Figure 8e

(35) $$Q\left(\overset{˘}{r}|r_{\mathrm{ex}}\right)=\frac{1}{3}\left[\begin{array}{cccccccccccccccccccc} 0 & 0 & 0 & 0 & 1 & 1 & 1 & 0 & 0 & 0 & 0 & 0 & 0 & 0 & 0 & 0 & 0 & 0 & 0 & 0\\ 0 & 0 & 0 & 0 & 0 & 1 & 1 & 1 & 0 & 0 & 0 & 0 & 0 & 0 & 0 & 0 & 0 & 0 & 0 & 0\\ 0 & 0 & 0 & 0 & 0 & 0 & 0 & 0 & 0 & 0 & 0 & 0 & 1 & 1 & 1 & 0 & 0 & 0 & 0 & 0\\ 0 & 0 & 0 & 0 & 0 & 0 & 0 & 0 & 0 & 0 & 0 & 0 & 0 & 1 & 1 & 1 & 0 & 0 & 0 & 0 \end{array}\right],$$

with rows and columns with the same convention as in Equation (34).

Finally, the noisy data (Figure 8a) can be obtained by transforming the degraded data (Figure 8d) with the transition matrix

(36) $$Q\left(r_{\mathrm{su}}|\overset{˘}{r}\right)=\frac{1}{3}\left[\begin{array}{cccccccccccccccccccc} 1 & 0 & 0 & 0 & 1 & 0 & 0 & 0 & 1 & 0 & 0 & 0 & 0 & 0 & 0 & 0 & 0 & 0 & 0 & 0\\ 0 & 1 & 0 & 0 & 0 & 1 & 0 & 0 & 0 & 1 & 0 & 0 & 0 & 0 & 0 & 0 & 0 & 0 & 0 & 0\\ 0 & 0 & 1 & 0 & 0 & 0 & 1 & 0 & 0 & 0 & 1 & 0 & 0 & 0 & 0 & 0 & 0 & 0 & 0 & 0\\ 0 & 0 & 0 & 1 & 0 & 0 & 0 & 1 & 0 & 0 & 0 & 1 & 0 & 0 & 0 & 0 & 0 & 0 & 0 & 0\\ 0 & 0 & 0 & 0 & 0 & 0 & 0 & 0 & 1 & 0 & 0 & 0 & 1 & 0 & 0 & 0 & 1 & 0 & 0 & 0\\ 0 & 0 & 0 & 0 & 0 & 0 & 0 & 0 & 0 & 1 & 0 & 0 & 0 & 1 & 0 & 0 & 0 & 1 & 0 & 0\\ 0 & 0 & 0 & 0 & 0 & 0 & 0 & 0 & 0 & 0 & 1 & 0 & 0 & 0 & 1 & 0 & 0 & 0 & 1 & 0\\ 0 & 0 & 0 & 0 & 0 & 0 & 0 & 0 & 0 & 0 & 0 & 1 & 0 & 0 & 0 & 1 & 0 & 0 & 0 & 1 \end{array}\right].$$

with rows and columns indicating the ordered sets {[1,2,3,4]×[2,4]} and {1,2,3,4}×{1,2,3,4,5}, respectively, where × denotes the Cartesian product with colexicographical order.

### 2.7. Conditions for Equality of the Amount and Type of Information Loss Reported by Different Measures

We now derive the conditions under which encoding/gray-oriented measures coincide with their decoding-oriented counterparts, as observed in Figure 2a and Figure 3d. That is, we derive the conditions under which the following equalities hold:

(37) $$\Delta I_{R_{\mathrm{su}}}=\Delta I_{}^{D}=\Delta I_{}^{DL},$$

(38) $$\Delta I_{\hat{S}}=\Delta I_{}^{LS},$$

(39) $$\Delta I_{\hat{S}}=\Delta I_{}^{B},$$

(40) $$\Delta A_{R_{\mathrm{su}}}=\Delta A_{}^{B}.$$

The example in Figure 7a showed that the existence of deterministic mappings does not suffice for a qualitative and quantitative equivalence of different measures. Furthermore, the example of Figure 3b showed that the equalities require the space of $R_{\mathrm{su}}$ to include the space of $R_{\mathrm{ex}}$, or else the decoding method α may be undefined. We demonstrate that the Equations (37)–(40) arise, and moreover, that there is no discrepancy in the type of information assessed by these different measures, whenever the mapping from $R_{\mathrm{ex}}$ into $R_{\mathrm{su}}$ can be described using positive-diagonal idempotent stochastic matrices [45]. Specifically, we prove the following theorem:

**Theorem** **5.** *Consider a stimulus-independent stochastic function f from a representation $R_{\mathrm{ex}}$ into another representation $R_{\mathrm{su}}$, such that the range R of $R_{\mathrm{su}}$ includes that of $R_{\mathrm{ex}}$, and with transition probabilities $Q(r_{\mathrm{su}}|r_{\mathrm{ex}})$ that can be written as positive-diagonal idempotent right stochastic matrices with row and column indices that enumerate the elements of R in the same order. Then, Equations (37)–(40) hold*.

**Proof.**  See Appendix B.6. □

The theorem states that the equalities of Equations (37)–(40) can be guaranteed whenever the removal of the tested response feature involves a (deterministic or) stochastic mapping $R_{\mathrm{ex}}\to R_{\mathrm{su}}$ that induces a partition within the set of real responses $R_{\mathrm{ex}}$, and $R_{\mathrm{su}}$ is obtained by rendering all responses inside each partition indistinguishable (but not across partitions). To sample $R_{\mathrm{su}}$, the probabilities of individual responses inside each partition are re-assigned, rendering their distinction uninformative [30].

This theorem provides sufficient but not necessary conditions for the equalities to hold. The important aspect, however, is that it ensures that the equalities hold not only in numerical value, but also, in the type of information that different measures ascribe to the tested feature. Two different methods preserve or lose information of different type if, when decoding a stimulus, the trials with decoding errors tend to confound different attributes of the stimulus, as in the example of Figure 8. The conditions of Theorem 5, however, ensure that the strategies α and β always decode exactly the same stimulus (see Appendix B.6), so there can be no difference in the confounded attributes. Pushing the argument further, one could even argue that responses (real or surrogate) encode more information than the identity of the stimulus that originated them. For a fixed decoded stimulus, the response still contains additional information [46], that refers to (a) the degree of certainty with which the stimulus is decoded, and (b) the rank of the alternative stimuli, in case the decoded stimulus was mistaken [20]. Both meanings are embodied in the whole rank of a posteriori probabilities $P_{\mathrm{su}}\left(s|r\right)$, not just the maximal one. Yet, under the conditions of the theorem, the entire rankings obtained with methods α and β coincide (see Appendix B.6). Therefore, even within this broader interpretation, there can be no difference in the qualitative aspects of the information preserved or lost by one and the other.

For example, in Figure 7b, we found that all information losses are equal (that is, $\Delta I_{\hat{R}}$, $\Delta I_{\hat{S}}$, $\Delta I_{\hat{S}}$, $\Delta I_{}^{D}$, $\Delta I_{}^{DL}$, $\Delta I_{}^{LS}$, and $\Delta I_{}^{B}$ are all 50%), and both accuracy losses are equal (that is, $\Delta A_{\hat{R}}$ and $\Delta A_{}^{B}$ are both ≈67%). However, the conditions of Theorem 5 do not hold. The matrix of Equation (32) is not block-diagonal, nor it can be taken to that shape by incorporating new rows (to make it square), and permuting both rows and columns, in such a way that the response vectors are enumerated in the same order by both indices. For this reason, the losses are not guaranteed to be of the same type.

Instead, the transition probabilities of Equations (15) and (16) can be turned into positive-diagonal idempotent right stochastic matrices. Equation (15) is already in the required format. To take Equation (16) to the conditions of Theorem 5, two new rows need to be incorporated, associated to the responses [4,1] and [2,2], that do not occur experimentally. Those rows can contain arbitrary values, since the condition $P_{\mathrm{ex}}\left(\left[4,1\right]|S\right)=P_{\mathrm{ex}}\left(\left[2,2\right]|S\right)=0,\;\forall S$ renders them irrelevant. Arranging the columns so that both rows and columns enumerate the same list of responses, Equation (16) can be written as

(41) $$Q\left(r_{\mathrm{su}}|r_{\mathrm{ex}}\right)=\left[\begin{array}{cccccc} b & \bar{b} & 0 & 0 & 0 & 0\\ b & \bar{b} & 0 & 0 & 0 & 0\\ 0 & 0 & c & \bar{c} & 0 & 0\\ 0 & 0 & c & \bar{c} & 0 & 0\\ 0 & 0 & 0 & 0 & d & \bar{d}\\ 0 & 0 & 0 & 0 & d & \bar{d} \end{array}\right],$$

with $R_{ex}=R_{su}=\left\{\left[2,1\right],\left[2,2\right],\left[3,1\right],\left[3,2\right],\left[4,1\right],\left[4,2\right]\right\}$. Hence, in these two examples, both the amount and type of information of encoding and decoding-based measures coincide.

### 2.8. Improving the Performance of Decoders Operating with Strategy α

In a previous paper [17], we demonstrated that neither $\Delta I_{}^{D}$ nor $\Delta I_{}^{DL}$ constitute lower bounds on the information loss induced by decoders constructed by disregarding the tested response feature. This means that some decoders may exist, that perform better than $D_{\mathrm{su}}\left(r\right)$ defined in Equation (6). In this section we discuss one possible way in which some of these improved decoders may be constructed, inspired in the example of Figure 8. Quite remarkably, the construction involves the addition of noise to the real responses, before feeding them to the decoder of Equation (6). Panel (a) shows a decoder constructed with noisy data ($R_{\mathrm{su}}$), and then employed to decode quality data ($R_{\mathrm{ex}}$; Figure 8b), thereby yielding information losses $\Delta I_{}^{D}=\Delta I_{}^{DL}=50\%$. These losses can be decreased by feeding the decoder with a degraded version $\overset{˘}{R}$ of the quality data (Figure 8d) generated through a stimulus-independent transformation that adds latency noise (Figure 8e). Decoding $R_{\mathrm{ex}}$ as if it were $R_{\mathrm{su}}$ by first transforming $R_{\mathrm{ex}}$ into $\overset{˘}{R}$ results in $\Delta I_{}^{D}=\Delta I_{}^{DL}\approx 33\%$, thereby recovering 33% of the information previously lost. On the contrary, adding spike-count noise will tend to increase the losses. Thus, adding suitable amounts and type of noise can increase the performance of approximate decoders, and the result is not limited to the case in which the response aspect is the amount of noise correlations. In addition, this result also indicates that, contrary to previously thought [47], decoding algorithms need not match the encoding mechanisms for performing optimally from an information-theoretical standpoint. All these results are a consequence of the fact that decoders operating with strategy α are not optimal, so it is possible to improve their performance by deterministic or stochastic manipulations of the response. In practice, our results open up the possibility of increasing the efficiency of decoders constructed with approximate descriptions of the neural responses, usually called approximate or mismatched decoders, by adding suitable amounts and types of noise to the decoder input.

## 3. Related Issues

### 3.1. Relation to Decomposition-Based Methods

Many measures of different types have been developed to assess how different response features of the neural code interact with each other. Some are based on direct comparisons between the information encoded by individual features, or collections of features (see for example [48,49,50], to cite just a few among many). Others distinguish between two or more potential dynamical models of brain activity [51], for example, by differentiating between conditional and unconditional correlations between neurons in the frequency domain [52]. Yet others, rely on decompositions or projections based on information geometry. In those, the mutual information between stimuli and responses $I_{R}$ is broken down as $I_{R}=\sum_{i}I_{R_{i}}^{\prime }+\mathrm{Synergy}\;\mathrm{Terms}+\mathrm{Redundancy}\;\mathrm{Terms}$, where $I_{R_{i}}^{\prime }$ represents the information contributed by the individual response feature $R_{i}$, and the remaining terms incorporate the synergy or redundancy between them. In the original approaches [53,54,55,56,57], the terms $I_{R_{i}}^{\prime }$ represented the information $I(R_{i};S)$ encoded in single response aspects irrespective of what be encoded in other aspects. In later studies, [58,59,60,61,62], these terms accounted for the information that is *only* encoded in individual aspects, taking care of excluding whatever be redundant with other aspects. The approach discussed in this paper is in the line of the studies Nirenberg et al. [8] and Schneidman et al. [9] and all their consequences. This line has some similarities and some discrepancies with the decomposition-based studies. We here comment on some of these relations.

- First, the measure $\Delta I_{R_{\mathrm{su}}}$ quantifies the relevance of a given feature with the difference $I_{R_{\mathrm{ex}}}-I_{R_{\mathrm{su}}}$. When the surrogate response $R_{\mathrm{su}}$ is equal to the original response $R_{\mathrm{ex}}$ with just a single component $R_{i}$ eliminated, $\Delta I_{R_{\mathrm{su}}}$ is equal to $I(R_{i};s|{\bar{R}}_{i})$, where ${\bar{R}}_{i}$ is the collection of all response aspects except $R_{i}$. In this case, $\Delta I_{R_{\mathrm{su}}}$ coincides with the sum of the unique and the synergistic contributions of the dual decompositions in the newest set of methods [63].
- Second, when assessing the relevance of a given response feature, we are often inclined to draw conclusions about the cost of ignoring the tested feature when aiming to decode the original stimulus. As shown in this paper, those conclusions depend not only on how stimuli are encoded, but also, on how they are decoded. The decomposition-based methods are mainly focused in the encoding problem, so they are less suited to draw conclusions about decoding.
- Finally, as discussed in Figure 8, not only the amount of (encoded or decoded) information matters, but also, what type. Decomposition-based methods, although not yet reaching a full consensus in their formulation, provide a valuable attempt to characterize how both the type and the amount of information is structured within the set of analyzed variables, in a way that is complementary to the present approach, specifically in analyzing the structure of the lattices obtained by associating different response features [58,63].

### 3.2. The Problem of Limited Sampling

Throughout the paper we assumed that the distribution $P_{\mathrm{ex}}\left(s,r\right)$ is known, or is accessible to the experimenter. In the examples, when we calculated information values, we plugged the true distributions into the formulas, without discussing the fact that such distribution may not be easily estimated with finite amounts of data. Whichever method is used to estimate $P_{\mathrm{ex}}\left(s,r\right)$, to a larger or lesser degree, the outcome is no more than an approximation. Hence, even $I_{R_{\mathrm{ex}}}$ (which is supposed to be the full information) is estimated approximately. Since $P_{\mathrm{su}}\left(s,r\right)$ is a modified version of $P_{\mathrm{ex}}\left(s,r\right)$, also $P_{\mathrm{su}}\left(s,r\right)$ can only be estimated approximately. Information measures, including Kullback-Leibler divergences, are highly sensitive to variations in the involved probabilities [20,32,64,65,66,67,68,69], and the latter are unavoidable in high-dimensional response spaces. The assessment of the relevance of a given feature, hence, requires experiments that contain sufficient samples so as to ensure that the correcting methods work. When the response space is large, the measures $\Delta I_{S}$, $\Delta I^{B}$ and the loss of accuracies are less sensitive to limited sampling than $\Delta I_{R_{\mathrm{su}}}$, $\Delta I^{D}$ and $\Delta I^{LD}$.

In addition, the problem of finite sampling can also be formulated as an attempt to determine the relevance of the feature “Accuracy in the estimation of $P_{\mathrm{ex}}\left(r|s\right)$”. This feature is not a property of the nervous system, but rather, of our ability to characterise it. Still, the framework developed here can also handle this methodological problem. The estimated distribution can be interpreted as a stochastic modification $P_{\mathrm{su}}\left(r|s\right)$ of the true distribution $P_{\mathrm{ex}}\left(r|s\right)$. As long as the caveats discussed in this paper are taken into account, the measures of Equations (21)–(29) may serve to evaluate the cost of modeling $P_{\mathrm{ex}}\left(r|s\right)$ out of finite amounts of data.

## 4. Conclusions

Several measures have been proposed in the literature to assess the relevance of specific response features in the neural code. All proposals are based on the idea that by removing the tested feature from the response, the neural code deteriorates, and the lost information is a useful measure of the relevance of the feature. In this paper, we demonstrated that the neural code may or may not deteriorate when removing a response feature, depending on the nature of the tested feature, and on the method of removal, in ways previously unseen. First, we determined the conditions under which the data processing inequality can be invoked. Second, we showed that decoding-oriented measures may result in larger or smaller losses than their encoding (or gray) counterparts, even for response aspects that, unlike noise correlations, can be modeled as stimulus-independent transformations of the full response. Third, we demonstrated that both types of measures coincide under the conditions of Theorem 5. Fourth, we showed that evaluating the role of a response feature in the neural code involves not only an assessment of its contribution to the amount of encoded information, but also, to the meaning of that information. Such meaning is as dependent as the amount on the measure employed to assess it. Finally, our results open up the possibility that simple and cheap decoding strategies, based on the addition of an adequate type and amount of noise, be more efficient and resilient than previously thought. We conclude that the assessment of the relevance of a specific response feature cannot be performed without a careful justification for the selection of a specific method of removal.

## Figures and Tables

**Table 1:** Numerical exploration of the maximum and minimum differences between several measures of information and accuracy losses. The values are expressed as percentages of $I_{R_{\mathrm{ex}}}$ (the information encoded in $R_{\mathrm{ex}}$) or $A_{R_{\mathrm{ex}}}^{R_{\mathrm{ex}}}$ (the maximum accuracy above chance level when decoders operate on $R_{\mathrm{ex}}$). All examples involve two stimuli, so $\Delta I_{\hat{S}}=\Delta I_{\hat{S}}$ and $\Delta I_{}^{LS}=\Delta I_{}^{B}$. The absolute value of $\Delta A_{R_{\mathrm{su}}}-\Delta A_{}^{B}$ can become extremely large when $A_{R_{\mathrm{su}}}^{R_{\mathrm{su}}}\approx 0$. Dashes represent cases in which decoding-oriented measures are undefined, as explained in Section 2.4.

Cases		Figure 4a	Figure 2b	Figure 2c	Figure 3d	Figure 2a	Figure 3b	Figure 7a
$\Delta I_{\hat{R}}-\Delta I_{}^{D}$	min	−79	−51	−34	0	0	—	≤999
	max	26	32	51	0	0	—	−20
$\Delta I_{\hat{R}}-\Delta I_{}^{DL}$	min	−34	−32	−16	0	0	−100	−100
	max	59	41	98	0	0	0	0
$\Delta I_{\hat{R}}-\Delta I_{}^{B}$	min	−67	−62	−46	−63	−87	—	−100
	max	57	81	96	0	0	—	0
$\Delta I_{\hat{S}}-\Delta I_{}^{D}$	min	−79	−48	−34	0	0	—	≤999
	max	67	92	93	63	87	—	70
$\Delta I_{\hat{S}}-\Delta I_{}^{DL}$	min	−34	−27	−16	0	0	−100	−100
	max	91	92	99	63	87	0	97
$\Delta I_{\hat{S}}-\Delta I_{}^{B}$	min	−51	−31	−17	0	0	—	−100
	max	59	91	98	0	0	—	100
$\Delta A_{\hat{R}}-\Delta A_{}^{B}$	min	−386	−200	−150	0	0	—	≤999
	max	95	67	100	0	0	—	0

## Appendix A. On the Information and Accuracy Differences

Each value in Table 1 (except for those associated with Figure 3b; see below) was computed using the Nelder-Mead simplex algorithm for optimization, as implemented by the function fminsearch of Matlab 2016. For accuracy reasons, only examples in which $I_{R_{\mathrm{ex}}}\geq 10^{-6}\;\mathrm{bits}$ and $A_{R_{\mathrm{ex}}}^{R_{\mathrm{ex}}}\geq 10^{-6}$ were considered. Furthermore, parameters defining the joint stimulus-response probabilities and the transition matrices were restricted to the interval [0.05,0.95]. Each difference between two measures defined in Equations (21)–(29) was computed repeatedly, with random initial values for the stimulus-response probabilities and the transition matrices, until the value of the difference failed to increase or decrease in 20 consecutive runs.

The values in Table 1 for Figure 3b were computed analytically with $P_{\mathrm{ex}}\left(\left[3,2\right]\right)>0$ or $P_{\mathrm{ex}}\left(\left[4,2\right]\right)>0$, but not both. In those cases, the measures $\Delta I_{}^{D}$, $\Delta I_{}^{B}$, and $\Delta A_{}^{B}$ are undefined, whereas $\Delta I_{}^{DL}=100\%$, for the reasons given in Section 2.4. However, $\Delta I_{R_{\mathrm{su}}}$ and $\Delta I_{\hat{S}}$ can vary between 0% and 100%, for example, attaining 0% when $P_{\mathrm{ex}}\left(\left[3,1\right]\right)\to 0$, and 100% when $P_{\mathrm{ex}}\left(\left[2,1\right]\right)\to 0$ and $P_{\mathrm{ex}}\left(\left[4,2\right]\right)\to 0$. The information $I_{R_{\mathrm{ex}}}$ equals the stimulus entropy, regardless of the response probabilities. The values in Table 1 for Figure 3d were computed by setting b=c=d=0.5 in Equation (16). The values in Section 2.4 for Figure 7b were obtained by setting $P_{\mathrm{ex}}\left(s,r\right)=1/4$ for the stimulus-response pairs shown in the figure, and are valid for any transition probability matrix set as in Equation (32) with b=a. The values in Section 2.4 for Figure 8 were obtained by setting $P_{\mathrm{ex}}\left(s,r\right)=1/4$ for the stimulus-response pairs shown in the figure.

## Appendix B. Proofs

### Appendix B.1. Derivation of Equation (30)

The definition of $\Delta I_{}^{DL}$ involves the probability $P_{\mathrm{su}}\left(s|\hat{r},\theta \right)$ defined in [11,36,38] as proportional to $P\left(S\right)\prod_{i}P^{\theta }\left(R_{i}|S\right)$, where the exponent θ is chosen so as to maximize $\Delta I_{}^{DL}$. This definition has been recently shown to be invalid when ∃r,s such that $P_{\mathrm{su}}\left(r|s\right)=0$ for a stimulus *s* or a response r for which $P_{\mathrm{ex}}\left(r|s\right)\neq 0$ [17]. This problem never appears when evaluating the relevance of noise correlations with $P_{\mathrm{su}}\left(r|s\right)=P_{NI}\left(r|s\right)$ as stated by Equation (17). Yet, it may well appear in more general cases, including those arising from stochastically reduced codes. To overcome it, we prove the theorem

**Theorem** **A1.**
*The probability P(s|r,θ) that appears in the definition of $\Delta I_{}^{DL}$ is*

$$P_{\mathrm{su}}\left(s|r,\theta \right)\propto \left\{\begin{array}{cc} P(s) & if\;\exists \hat{s},\hat{r}\;such\;that\;P_{\mathrm{ex}}\left(\hat{r}|\hat{s}\right)>P_{\mathrm{su}}\left(\hat{r}|\hat{s}\right)=0\\ 0 & if\;P_{\mathrm{su}}\left(r|s\right)=P_{\mathrm{ex}}\left(r|s\right)=0\;for\;some\;but\;not\;all\;s\\ P\left(s\right)P_{\mathrm{su}}{\left(r|s\right)}^{\theta } & otherwise \end{array}\right.$$

**Proof.** According to Latham and Nirenberg [11], the probability $P_{\mathrm{su}}\left(s|r,\theta \right)$ is the one that minimizes the Kullback-Leibler divergence $D_{\mathrm{KL}}[P^{*}\left(r,s\right)||p\left(r\right)p\left(s\right)]$ with respect to the distribution $P^{*}\left(r,s\right)$, subject to the constraints

(A1) $$\begin{array}{c} {\langle \log_{2}P_{\mathrm{su}}\left(r|s\right)\rangle }_{P^{*}\left(r,s\right)}={\langle \log_{2}q\left(r|s\right)\rangle }_{P(s,r)} \end{array}$$

(A2) $$\begin{array}{c} \sum_{s}P^{*}\left(r,s\right)=P(r). \end{array}$$

The minimization problem can be formulated in terms of an objective function to be minimized, in which the constraints appear with Lagrange multipliers, and θ is the one accompanying Equation (A1). Using the standard conventions that 0log0=0 and xlog0=∞ for x>0, Equation (A1) is fulfilled if $\exists \;\hat{r},\hat{s}$ such that $P(\hat{s}|\hat{r},\theta )>0$ if $P_{\mathrm{ex}}\left(\hat{r},\hat{s}\right)>P_{\mathrm{su}}\left(\hat{r}|\hat{s}\right)=0$. The first part of the theorem immediately follows by solving Equation (B15) in [11] as there indicated with β=0. If $∄\;\hat{r},\hat{s}$ such that $P_{\mathrm{ex}}\left(\hat{r},\hat{s}\right)>P_{\mathrm{su}}\left(\hat{r}|\hat{s}\right)=0$, then Equation (A1) is fulfilled only if P(s,r|θ)=0 when $P_{\mathrm{su}}\left(r|s\right)=P_{\mathrm{ex}}\left(r,s\right)=0$. The second and third parts of the theorem immediately follows using Bayes’ rule. □

### Appendix B.2. Proof of Theorem 1

**Proof.** The second part is proved by the two examples in Figure 4. The first part was proved in [9], at least for cases in which the set of the surrogate responses $R_{\mathrm{su}}=R_{NI}$ differ from the set of the real responses $R_{\mathrm{ex}}$. When they both coincide, we can prove the first part by contradiction, assuming that a deterministic mapping exists from $R_{\mathrm{ex}}$ into $R_{NI}$. If both variables sample the same response space, the deterministic mapping must be one-to-one, otherwise the variable $R_{NI}$ would sample a smaller set. Therefore, both $R_{NI}$ and $R_{\mathrm{ex}}$ maximize the conditional entropy given *S* over the probability distributions with the same marginals, since one-to-one mappings do not modify the entropy, and $R_{NI}$ is defined as the distribution with maximal conditional entropy with fixed marginals. Because the probability distribution achieving this maximum is unique [16], $P_{\mathrm{su}}\left(r|s\right)$ and $P_{\mathrm{ex}}\left(r|s\right)$ must be the same, thereby proving the theorem. □

### Appendix B.3. Proof of Theorem 2

**Proof.** We prove the dependency on the marginal likelihoods by computing $Q(r_{\mathrm{su}}|r_{\mathrm{ex}})$ for the hypothetical experiment of Figure 4a, and observing that the result depends on the marginal likelihood $P_{\mathrm{ex}}\left(L|s\right)$. To that end, we rewrite Equation (10) for $R_{\mathrm{su}}=\left[1,2\right]$ as

$$P_{\mathrm{su}}\left(\left[1,2\right]|□\right)=P_{\mathrm{ex}}\left(\left[1,2\right]|□\right)Q\left(\left[1,2\right]|\left[1,2\right]\right)+P_{\mathrm{ex}}\left(\left[2,1\right]|□\right)Q\left(\left[1,2\right]|\left[2,1\right]\right).$$

Note that $P_{\mathrm{ex}}\left(\left[1,2\right]|□\right)=1-P_{\mathrm{ex}}\left(\left[2,1\right]|□\right)=P_{\mathrm{ex}}\left(L=1|□\right)$ and $P_{\mathrm{su}}\left(\left[1,2\right]|□\right)=P_{\mathrm{ex}}{\left(L=1|□\right)}^{2}$. Using this and rearranging the terms, we obtain the quadratic equation

$$P_{\mathrm{ex}}{\left(L=1|□\right)}^{2}+P_{\mathrm{ex}}\left(L=1|□\right)\left\{Q([1,2]|[2,1])-Q([1,2]|[1,2])\right\}-Q\left(\left[1,2\right]|\left[2,1\right]\right)=0,$$

that is solved by

$$P_{\mathrm{ex}}\left(L=1|□\right)=0.5\left[\delta q+{\left(\delta q^{2}+4Q\left(\left[1,2\right]|\left[2,1\right]\right)\right)}^{0.5}\right],$$

where δq=Q([1,2]|[1,2])−Q([1,2]|[2,1]). Hence, any change in $P_{\mathrm{ex}}\left(L=1|□\right)$ must be followed by some change in $Q(r_{\mathrm{su}}|r_{\mathrm{ex}})$, thereby proving the first part. We prove the dependency on the noise correlations by computing $Q(r_{\mathrm{su}}|r_{\mathrm{ex}})$ for the hypothetical experiment of Figure 5, and observing that the result not only depends on the marginal likelihoods $P_{\mathrm{ex}}\left(L|s\right)$ and $P_{\mathrm{ex}}\left(C|s\right)$, but in many cases, it also depends on the joint distributions $P_{\mathrm{ex}}\left(L,C|s\right)$. Hence, varying the amount of noise correlations, even if keeping the marginals fixed, yields a variation in the mapping $Q(r_{\mathrm{su}}|r_{\mathrm{ex}})$. We proceed by reductio ad absurdum. If $Q(r_{\mathrm{su}}|r_{\mathrm{ex}})$ does not depend on the amount of noise correlations in $P_{\mathrm{ex}}\left(r|s\right)$, we may assume that if we vary $P_{\mathrm{ex}}\left(r|s\right)$ but keep the marginals $P_{\mathrm{ex}}\left(r_{i}|s\right)$ fixed, the transition probabilities $Q(r_{\mathrm{su}}|r_{\mathrm{ex}})$ remain unchanged. Under this hypothesis, Equation (10) is valid for many choices of $P_{\mathrm{ex}}\left(r|s\right)$. In this context, consider the set of all response distributions with the same marginals as $P_{\mathrm{ex}}\left(r|s\right)$ that can be turned into $P_{\mathrm{su}}\left(r|s\right)$ through $Q(r_{\mathrm{su}}|r_{\mathrm{ex}})$. This set includes $P_{\mathrm{su}}\left(r|s\right)$, and therefore, $Q(r_{\mathrm{su}}|r_{\mathrm{ex}})$ should be able to transform $P_{\mathrm{su}}\left(r|s\right)$ into itself. In addition, Property 2 requires that $Q(r_{\mathrm{su}}|\left[2,2\right])=0$ when $r_{\mathrm{su}}\neq \left[2,2\right]$ because either $P(r_{\mathrm{su}}|□)=0$ or $P(r_{\mathrm{su}}|◯)=0$ for those responses. Normalization yields Q([2,2]|[2,2])=1. Furthermore, computing Equation (10) for $R_{\mathrm{su}}=\left[2,2\right]$ yields

$$0=P_{\mathrm{ex}}\left(\left[1,1\right]|□\right)Q\left(\left[2,2\right]|\left[1,1\right]\right)+P_{\mathrm{ex}}\left(\left[1,2\right]|□\right)Q\left(\left[2,2\right]|\left[1,2\right]\right)+P_{\mathrm{ex}}\left(\left[2,1\right]|□\right)Q\left(\left[2,2\right]|\left[2,1\right]\right),$$

which shows that $Q(\left[2,2\right]|r_{\mathrm{ex}})=0$ when $r_{\mathrm{ex}}\in \left\{\left[1,1\right],\left[1,2\right],\left[2,1\right]\right\}$. Consequently, the resulting $Q(r_{\mathrm{su}}|r_{\mathrm{ex}})$ yields through Equation (10) that $P_{\mathrm{su}}\left(\left[2,2\right]|□\right)=P_{\mathrm{ex}}\left(\left[2,2\right]|□\right)$. After noticing that

$$P_{\mathrm{su}}\left(\left[2,2\right]|□\right)=P_{\mathrm{ex}}\left(L=2|□\right)P_{\mathrm{ex}}\left(C=2|□\right),$$

and that

$$P_{\mathrm{ex}}\left(\left[2,2\right]|□\right)=P_{\mathrm{ex}}\left(L=2|□\right)-P_{\mathrm{ex}}\left(\left[1,2\right]|□\right)=P_{\mathrm{ex}}\left(C=2|□\right)-P_{\mathrm{ex}}\left(\left[2,1\right]|□\right),$$

we can show that, after some straightforward algebra, Equation (10) only holds if $P_{\mathrm{su}}\left(r|□\right)=P_{\mathrm{ex}}\left(r|□\right)$ for all r. Thus, the initial hypothesis yields a transition matrix $Q(r_{\mathrm{su}}|r_{\mathrm{ex}})$ that is unable to transform $R_{\mathrm{ex}}$ into $R_{\mathrm{su}}$ when $R_{\mathrm{ex}}$ is noise correlated, and thus $Q(r_{\mathrm{su}}|r_{\mathrm{ex}})$ necessarily depends on the amount of noise correlations in $R_{\mathrm{ex}}$. □

### Appendix B.4. Proof of Theorem 3

**Proof.** The condition $\Delta I^{D}=\Delta I_{R_{\mathrm{su}}}$ implies that

(A3) $$\sum_{sr}P_{\mathrm{ex}}\left(s,r\right)\log\left[\frac{P_{\mathrm{ex}}\left(s|r\right)}{P_{\mathrm{su}}\left(s|r\right)}\right]=I_{R_{\mathrm{ex}}}-I_{R_{\mathrm{su}}}.$$

However,

$$\Delta I^{D}=I_{R_{\mathrm{ex}}}-\sum_{sr}P_{\mathrm{ex}}\left(s,r\right)\log\left[\frac{P_{\mathrm{su}}\left(r|s\right)}{P_{\mathrm{su}}\left(r\right)}\right].$$

Hence, Equation (A3) becomes

(A4) $$-\sum_{sr}P_{\mathrm{ex}}\left(s,r\right)\log\left[\frac{P_{\mathrm{su}}\left(r|s\right)}{P_{\mathrm{su}}\left(r\right)}\right]=-I_{R_{\mathrm{su}}}$$

In addition, when evaluating the relevance of noise correlations, $P_{\mathrm{su}}\left(r,s\right)=P\left(s\right)P_{NI}\left(r|s\right)$ as established by Equation (17). Hence,

(A5) $$\begin{array}{c} -\sum_{sr}P_{\mathrm{ex}}\left(s,r\right)\log\left[\frac{P_{\mathrm{su}}\left(r|s\right)}{P_{\mathrm{su}}\left(r\right)}\right]=\sum_{j}H\left(R_{j}|s\right)+\sum_{sr}P_{\mathrm{ex}}\left(r,s\right)\log\left[P_{\mathrm{su}}\left(r\right)\right] \end{array}$$

(A6) $$\begin{array}{c} -I_{R_{\mathrm{su}}}=\sum_{j}H\left(R_{j}|s\right)+\sum_{sr}P_{\mathrm{su}}\left(s,r\right)\log\left[P_{\mathrm{su}}\left(r\right)\right]. \end{array}$$

Replacing Equations (A5) and (A6) in Equation (A4),

$$\sum_{sr}P_{\mathrm{ex}}\left(s,r\right)\log\left[P_{\mathrm{su}}\left(r\right)\right]=\sum_{sr}P_{\mathrm{su}}\left(s,r\right)\log\left[P_{\mathrm{su}}\left(r\right)\right].$$

Summing in *s*, and rearranging,

$$\sum_{r}\left[P_{\mathrm{ex}}\left(r\right)-P_{\mathrm{su}}\left(r\right)\right]\log\left[P_{\mathrm{su}}\left(r\right)\right]=0.$$

If instead of an equality, we start with an inequality, that same inequality can be kept all through the proof. □

### Appendix B.5. Proof of Theorem 4

**Proof.** Consider a neural code $R_{\mathrm{ex}}=\left[R_{1},\ldots ,R_{N}\right]$ and recall that the range of $R_{NI}$ includes that of $R_{\mathrm{ex}}$. Therefore, $\Delta I_{}^{DL}=I_{R_{\mathrm{ex}}}$ implies that the minimum in Eqution (26) is attained when θ=0. In that case, Equation (B13a) in [11] yields

$$\sum_{s,r_{n}}P\left(s,r_{n}\right)\log_{2}P\left(r_{n}|s\right)=\sum_{s,r_{n}}P\left(s\right)P_{\mathrm{ex}}\left(r_{n}\right)\log_{2}P_{\mathrm{ex}}\left(r_{n}|s\right),\;\forall \;1\leq n\leq N,\;\forall \;n.$$

After some more algebra and recalling that the Kullback-Leibler divergence is never negative, this equation becomes $I_{R_{n}}=0$, implying that when read isolatedly, single responses contain no information about the stimulus. Consequently $\Delta I_{R_{NI}}=I_{R_{\mathrm{ex}}}$, thereby proving the “only if” part. For the “if” part, it is sufficient to notice that the last equality implies that $P_{NI}\left(r|s\right)=P_{NI}\left(r\right)$. □

### Appendix B.6. Proof of Theorem 5

**Proof.** The conditions on *f* and $Q(r_{\mathrm{su}}|r_{\mathrm{ex}})$ ensure that $Q(r_{\mathrm{su}}|r_{\mathrm{ex}})$ can be written as a block-diagonal matrix, each block composed of the same rows with no zeros, and that each block can be associated with a non-overlapping partition $R_{1},\ldots ,R_{M}$ of the range of *f*. Under these conditions, $P\left(r_{\mathrm{su}}|r_{\mathrm{ex}}\right)=P\left(r_{\mathrm{su}}|R_{m}\right)$ when $r_{\mathrm{ex}}\in R_{m}$. Hence, for $r_{\mathrm{su}}\in R_{m}$, $P\left(r_{\mathrm{su}}|s\right)=P\left(r_{\mathrm{su}}|R_{m}\right)P\left(R_{m}|s\right)$, yielding $P\left(s|r_{\mathrm{su}}\right)=P\left(s|R_{m}\right)$ and $P\left(s|r_{\mathrm{su}},\theta \right)=P\left(s|R_{m},\theta \right)$. Recomputing Equations (21)–(29) with these equalities in mind immediately yields the equalities in the theorem. Even when the amount of information is equal, differences in the type of information may arise because the measures are based on different decoding strategies, here denoted α and β. However, under the conditions of the theorem, decoding strategy α and decoding strategy β are one and the same. Because $P\left(s|r_{\mathrm{su}}\right)=P\left(s|R_{m}\right)$, both decoding strategies choose *s* only based on the partition R of $r_{\mathrm{ex}}$ or $r_{\mathrm{su}}$, respectively. Mathematically, both choose *s* according to

$$\hat{s}=\arg\max_{s}P(s|R(r)),$$

where R(r) denotes the mapping from r into R, which is the same regardless of whether r is $r_{\mathrm{ex}}$ or $r_{\mathrm{su}}$. Because $Q(r_{\mathrm{su}}|r_{\mathrm{ex}})$ maps each partition onto itself, the responses within each partition of $r_{\mathrm{su}}$ is completely generated by the responses in each partition of $r_{\mathrm{ex}}$, and thus the decoding strategies are applied to the same set of $r_{\mathrm{ex}}$. Hence, both decoding strategies are defined and operate in the same manner, yielding the same information. □

## Appendix C. On the Computation of Δ*I^D^*

The information loss caused by mismatched decoders (decoding strategy α) when $R_{\mathrm{su}}=f\left(R_{\mathrm{ex}}\right)$ has previously been computed as $\Delta I_{}^{D}$ but with $P_{\mathrm{su}}\left(s|r\right)$ replaced by $P\left(s|R_{\mathrm{su}}=f\left(r\right)\right)=P_{\mathrm{su}}\left(s|f\left(r\right)\right)$ [10,12,13]. The latter represents the probability of *s* given that $R_{\mathrm{su}}$ takes the value f(r), thereby limiting *f* to deterministic mappings. However, the probabilities $P_{\mathrm{su}}\left(s|r\right)$ and $P_{\mathrm{su}}\left(s|f\left(r\right)\right)$ are not equivalent, since

$$\begin{array}{cc} P_{\mathrm{su}}\left(s|r\right) & \propto \sum_{r=f(\hat{r})}P_{\mathrm{ex}}\left(\hat{r},s\right)\\ P_{\mathrm{su}}\left(s|f\left(r\right)\right) & \propto \sum_{f\left(r\right)=f\left(\hat{r}\right)}P_{\mathrm{ex}}\left(\hat{r},s\right) \end{array}$$

These two definitions raise the question of which alternative is the appropriate one when computing the information loss caused by mismatched decoders.

To resolve this question, notice that replacing $P_{\mathrm{su}}\left(s|r\right)$ with $P_{\mathrm{su}}\left(s|f\left(r\right)\right)$ in Equation (6) yields the decoding algorithm

$$\hat{s}=\arg\max_{s}P_{\mathrm{su}}\left(s|f\left(r\right)\right).$$

This algorithm entails first transforming the observed r into $r_{\mathrm{su}}=f\left(r\right)$, and then choosing the stimulus $\hat{s}=D_{\mathrm{su}}\left(\hat{r}\right)$ with a matched probability. Hence, its operation is analogous to the decoding algorithm β, and not, as originally intended, to the decoding algorithm α.

To illustrate the difference, recall the experiment in Figure 7a and suppose that the observed response is r=0.25π. The decoding algorithm α reads this value, computes $P_{\mathrm{su}}\left(s|0.25\pi \right)$, and decodes $\hat{s}=□$. Instead, the decoding algorithm proposed in [10,12,13], first transforms the value of r=0.25π into f(r)=0.75π, then computes $P_{\mathrm{su}}\left(s|0.75\pi \right)$, and finally decodes $\hat{s}=◯$. This mode of operation corresponds to the decoding algorithm β.

The above discrepancy can also be seen from the change in the operational meaning of $\Delta I_{}^{D}$ caused by the replacement. To that end, recall that $\Delta I_{}^{D}$ was first introduced as a comparison between the average number of binary questions required to identify *s* after observing r when using two optimal question-asking strategies, one tailored for $P_{\mathrm{ex}}\left(s|r\right)$ and the other for $P_{\mathrm{su}}\left(s|r\right)$ [8]. Mathematically, this difference can be written as

(A7) $$\Delta I_{}^{D}=\sum_{s,r}P_{\mathrm{ex}}\left(s,r\right)\log_{2}P_{\mathrm{ex}}\left(s|r\right)-\sum_{s,r}P_{\mathrm{ex}}\left(s,r\right)\log_{2}P_{\mathrm{su}}\left(s|r\right).$$

In each term, the argument of the logarithms is determined by the question-asking strategy, whereas the weight of the averages is determined by the probability distribution of the variables on which the strategy is applied [8,10,16]. Equation (A7) describes the decoding strategy α.

Replacing $P_{\mathrm{su}}\left(s|r\right)$ with $P_{\mathrm{su}}\left(s|f\left(r\right)\right)$ turns Equation (A7) into

$$\begin{array}{cc} \Delta \tilde{I} & =\sum_{s,r}P_{\mathrm{ex}}\left(s,r\right)\log_{2}P_{\mathrm{ex}}\left(s|r\right)-\sum_{s,r}P_{\mathrm{ex}}\left(s,r\right)\log_{2}P(s|f(r))\\ & =\sum_{s,r}P_{\mathrm{ex}}\left(s,r\right)\log_{2}P_{\mathrm{ex}}\left(s|r\right)-\sum_{s,r_{\mathrm{su}}}P_{\mathrm{su}}\left(s,r_{\mathrm{su}}\right)\log_{2}P_{\mathrm{su}}\left(s|r_{\mathrm{su}}\right)\\ & =\Delta I_{R_{\mathrm{su}}}. \end{array}$$

Unlike $\Delta I_{}^{D}$, this difference compares the average number of binary questions required to identify *s* after observing r using a question-asking strategy that is optimal for $P_{\mathrm{ex}}\left(s|r\right)$, with the average number of binary questions required to identify *s* after observing $r_{\mathrm{su}}$ using the a question-asking strategy that is optimal for $P_{\mathrm{su}}\left(s|r_{\mathrm{su}}\right)$. This is the way the decoding strategy β operates, not α.

Naively, one may think that a change in $P_{\mathrm{su}}\left(s|r\right)$, regardless of its size, may turn the measure $\Delta I_{R_{\mathrm{su}}}$, typically regarded as an encoding-oriented measure and here linked to the decoding algorithm β, into the decoding-oriented measure $\Delta I_{}^{D}$. However, notice that this change cannot occur through the equations above due to the change induced in $P_{\mathrm{su}}\left(s,r_{\mathrm{su}}\right)$. For that to actually occur, one must write $\Delta I_{R_{\mathrm{su}}}$ differently, as for example:

$$\Delta I_{R_{\mathrm{su}}}=\sum_{s,r}P_{\mathrm{ex}}\left(s,r\right)\log_{2}P_{\mathrm{ex}}\left(s|r\right)-\sum_{s,r_{\mathrm{su}}}P_{\mathrm{ex}}\left(s,r\right)\log_{2}P_{\mathrm{su}}\left(s|r_{\mathrm{su}}\right).$$

In this reformulation, the second term can be interpreted as the average number of binary questions required to identify *s* after observing r using a question-asking strategy that is optimal for $P_{\mathrm{su}}\left(s|r_{\mathrm{su}}\right)$, but only after converting r into $r_{\mathrm{su}}$. Any change in $P_{\mathrm{su}}\left(s|r_{\mathrm{su}}\right)$ immediately renders $P_{\mathrm{su}}\left(s|r_{\mathrm{su}}\right)$ a mismatched probability for $r_{\mathrm{su}}$, and makes the second term represent the average number of binary questions required to identify *s* after observing r using the question-asking strategy that is optimal for an altered version of $P_{\mathrm{su}}\left(s|r_{\mathrm{su}}\right)$ but only after converting r into $r_{\mathrm{su}}$, which need not resemble the meaning of the second term in $\Delta I_{}^{D}$.
